# Artificial Intelligence Literacy and the Responsible Use of Generative AI Among University Students in Peru: The Mediating Role of Self-Regulated Learning and the Moderating Effect of Technostress

**DOI:** 10.3390/ejihpe16090143

**Published:** 2026-09-17

**Authors:** Sandra Lizzette León Luyo, Heyner Yuliano Marquez-Yauri, Christian David Corrales Otazú, Sarita Jessica Apaza Miranda, César Pol Arévalo-Aranda, Marcos Marcelo Flores Castillo, Luisa Angélica Orejuela Guerrero, César Augusto Guzmán Valle, Marco Agustín Arbulú Ballesteros, Ana Elizabeth Paredes Morales

**Affiliations:** 1 Facultad de Ciencias Económicas, Escuela de Administración, Universidad Nacional de Trujillo, Trujillo 13011, Peru; saleon@unitru.edu.pe (S.L.L.L.); hmarquez@unitru.edu.pe (H.Y.M.-Y.); 2 Facultad de Ciencias Jurídicas y Políticas, Escuela de Derecho, Universidad Católica de Santa María, Arequipa 04013, Peru; 3 Facultad de Ciencias de la Empresa, Escuela de Administración y Negocios Internacionales, Universidad Continental, Campus Arequipa, Arequipa 04001, Peru; sapaza@continental.edu.pe; 4 Departamento Académico de Ingeniería Metalúrgica, Escuela Profesional de Ingeniería Metalúrgica, Universidad Nacional de Trujillo, Trujillo 13011, Peru; carevalo@unitru.edu.pe; 5 Escuela de Administración, Facultad de Ciencias Sociales y Empresariales, Universidad Nacional de Frontera, Sullana 20101, Peru; mflores@unf.edu.pe; 6 Facultad de Ingeniería, Escuela Profesional de Ingeniería Industrial, Universidad Nacional de Trujillo, Trujillo 13011, Peru; lorejuela@unitru.edu.pe; 7 Departamento de Ingeniería de Sistemas, Universidad Nacional Pedro Ruiz Gallo, Lambayeque 14013, Peru; cguzmanv@unprg.edu.pe; 8 Institute for Research in Science and Technology, César Vallejo University, Campus Chepén, Trujillo 13001, Peru; marbulub@ucv.edu.pe (M.A.A.B.); aparedesm@ucv.edu.pe (A.E.P.M.)

**Keywords:** AI literacy, responsible use of generative AI, self-regulated learning, technostress, moderated mediation, higher education, Peru

## Abstract

University students’ adoption of generative artificial intelligence has become nearly universal, yet institutional guidance has not kept pace, leaving responsible use dependent on students’ own self-regulation. Drawing on social cognitive theory, this study examined whether self-regulated learning accounts for the association between AI literacy and self-reported responsible use of generative AI, and how technostress relates to that association. A cross-sectional survey of 820 Peruvian undergraduates from public and private universities was analyzed using covariance-based structural equation modeling; a two-stage moderated mediation model with latent interactions was estimated using latent moderated structural equations. Measurement invariance held across gender and university type, and the available diagnostics did not indicate a dominant common-method factor. AI literacy predicted self-regulated learning (β = 0.626) and responsible use (β = 0.243), and self-regulated learning predicted responsible use (β = 0.445). The indirect effect was significant, β = 0.278, 95% CI [0.226, 0.337], accounting for 53.4% of the total effect. Technostress showed no significant moderation of the first stage or the direct path, but the second-stage path was significantly weaker at higher levels of technostress (*b*_2_ = −0.211, *p* < 0.001): the conditional indirect effect was 0.344 at low and 0.111 at high technostress and was not statistically distinguishable from zero above the estimated region-of-significance boundary of +1.40 SD. Technostress was not associated with weaker self-regulation, but with a weaker association between self-regulation and its behavioural expression. Because the design is cross-sectional, all estimates are reported as associations.

## 1. Introduction

The adoption of generative artificial intelligence among college students went from being used by a minority to nearly universal in three years. The Higher Education Policy Institute’s annual series reports that the proportion of British students using it in graded assignments rose from 53% in 2024 to 88% in 2025 and to 94% in 2026, based on samples of over 1000 cases in each measurement (Freeman, 2025; Stephenson & Armstrong, 2026). In Latin America, the scale is comparable: a survey of 22,941 students from 29 institutions in twelve countries, including Peru, found that 92% actively use these tools, and 67% do so daily or weekly (Digital Education Council, 2026). What failed to keep pace with this trend was the institutional framework. In the same 2026 British survey, only 36% of students feel encouraged by their university to use generative AI, and just 38% report that their institution provides it to them. The relevant question for university policy is no longer whether students will use it, but what ensures they use it responsibly when no one has explained how to them.

This gap is particularly evident in Peru. In 2021, approximately 1.3 million students attended 92 accredited universities in Peru. As of the third quarter of 2025, 95.8% of Peruvians aged 19 to 24 were internet users, the highest rate among all age groups (National Superintendency of Higher Education, 2021; National Institute of Statistics and Informatics, 2025). Although they had internet access, no specific guidelines were provided. Montoya-Cantoral et al. (2023) noted a lack of Peruvian university regulations regarding the use of intellectual property and artificial intelligence (AI). At a public university in Peru’s Andean region, Oré Gálvez et al. (2026) demonstrated how a lack of regulation leads to a discrepancy between the use of AI that students acknowledge privately and the use of AI that they are permitted to engage in. Thus, students control their own visibility regarding AI use, as they are unsure of what constitutes acceptable AI use in the eyes of faculty. In the absence of regulation, the responsible use of AI is no longer an obligation, but rather something students can control.

This study examines this shift toward self-regulation. Social cognitive theory holds that behavior does not automatically follow from competence: self-observation, evaluation against personal standards, and subsequent adjustment mediate the gap between knowing and acting (Bandura, 1986, 1991), processes that, in the academic domain, are organized within Zimmerman’s (2000) cycle of anticipation, execution, and self-reflection. Applied to this case, artificial intelligence literacy, understood as the ability to recognize, use, evaluate, and ethically assess these systems (B. Wang et al., 2023), would be the enabling competency; self-regulated learning is the proposed mechanism through which that competence would be associated with responsible use; that is, adherence to academic rules, verification of generated content, and transparent attribution of authorship (Chan, 2023; UNESCO, 2023). There is evidence at both ends of this continuum. A three-level meta-analysis of 95 effect sizes from 28 studies confirms that AI interventions improve self-regulation (J. Wu et al., 2026), and a quasi-experiment showed that explicit metacognitive support enhances task strategy and self-assessment in generative environments, while its absence impairs them (Xu et al., 2025). The intermediate link is missing. No study has examined whether self-regulation accounts for the association between competence and behavior.

Nor does the chain operate in an emotional vacuum. Technostress, defined within the theory of person–environment mismatch as the tension that arises when technological demands exceed available capacities or when the resources provided do not meet the user’s needs (Edwards et al., 1998; Ragu-Nathan et al., 2008), consumes the very cognitive resources that self-regulation needs to sustain itself. Resource conservation theory predicts that, when faced with the threat of loss, people protect their reserves and cut back on investment in costly activities (Hobfoll, 1989). That generative systems act on precisely the resource at stake in this argument is documented directly: among 813 university students, the motivational features of generative AI were associated with learning outcomes and academic resilience through the reduction in cognitive load, which establishes cognitive load as the currency through which these tools reach the learner (Rehman et al., 2026). Technological mismatch is expected to deplete that same currency, so the two studies describe opposite movements along a single dimension rather than unrelated phenomena. Generating an output and declaring its use are costly activities. The available evidence on technostress and AI in higher education is scarce and inconsistent: among 649 Chinese students, technostressors related to generative AI affected learning performance through self-efficacy, with varying patterns depending on the stressor (Yang & Tang, 2026); among 876 pre-service teachers at twelve Peruvian universities, stress mediated the relationship between academic overload and the use of AI models (Acosta-Enriquez et al., 2025b).

The gap addressed by this study is best understood by examining what Peruvian research has accomplished and what remains to be done. The available studies establish bivariate associations or single-path models: academic self-efficacy is inversely correlated with AI dependence among 186 students (Estrada-Araoz et al., 2025); self-efficacy and anxiety predict that dependence among 528 students (Morales-García et al., 2025); attitudes toward AI influence academic ethics among 406 students at six universities (Espinoza Vidaurre et al., 2025); digital competencies and AI use predict engagement among 372 university students in Lima, with a negative correlation for AI use (Flores Huamancuri & Jaimes Marcacuzco, 2026). Two studies go so far as to name the mechanism of interest here without modeling it. Vereau Amaya et al. (2025) concludes, based on a sample of 200 first-year students in Trujillo, that unmediated use of AI could displace the self-regulatory processes of new students. García Castro et al. (2026) describe, based on 265 interviews in Tacna, a shift from instrumental efficiency toward cognitive delegation and a decline in critical autonomy. None of them examine self-regulation as a mediator.

The case of technostress is more telling. Marquez-Yauri et al. (2026) tested among 340 Peruvian college students whether technostress moderates the relationship between self-efficacy in AI and critical thinking and found no support: the direct effect of technostress was null (β = −0.06, *p* = 0.50), and the moderation did not survive a strict bootstrap test. The authors concluded that technostress is, at most, a distal correlate. Their design, however, did not allow for ruling out an alternative interpretation: that the moderator was well chosen but misplaced. If technostress does not prevent students from acquiring competence or reasoning better but does make it more burdensome to pause to verify information and claim authorship, its effect should not be sought in the first stage of the chain but in the last, where self-regulation would have to be expressed in behavior. That hypothesis, which relocates the moderator to the second stage of a mediated process, remains untested. Added to the gaps is one related to measurement, because the responsible use of generative AI lacks a validated scale: the closest Peruvian instrument covers regulatory compliance and writing support, without addressing content verification or authorship transparency (Garro-Aburto et al., 2026). There is also a methodological gap, as the cited studies estimate models based on observed scores, using samples ranging from 150 to 876 cases, without correcting for measurement error or specifying interactions among latent variables.

The present study aims to determine the extent to which artificial intelligence literacy predicts the responsible use of generative AI among Peruvian college students, to establish whether self-regulated learning mediates this relationship, and to identify the stage of the model at which technostress moderates its magnitude. Three research questions guide this study. Does self-regulated learning mediate the effect of AI literacy on responsible use, and to what extent? Does technostress moderate the association between AI literacy and self-regulation, the association between self-regulation and responsible use, or neither? Across what range of technostress is the indirect association statistically distinguishable from zero? To answer these questions, a two-stage moderated mediation model with latent variables was estimated using data from 820 students at public and private universities in Lima and other regions of the country.

This study contributes on three fronts. It shifts the discussion on AI literacy from describing levels to examining a proposed mechanism, by testing whether the data are consistent with the social-cognitive premise that competence requires self-regulatory agency to translate into behavior. It provides an empirical response to the ambiguity left open by Peruvian evidence on technostress, identifying the stage of the model at which moderation by technological stress is detected, rather than treating it as an undifferentiated main effect. It also offers a three-dimensional operationalization of the responsible use of generative AI, distinguishing between normative integrity, content verification, and authorship, all subjected to the reliability, validity, and invariance tests required by the psychometric literature. All of this takes place in a context where the absence of institutional norms makes student self-regulation the primary resource available.

The article begins with a literature review and the development of hypotheses, continues with the methodology, presents the results of the measurement model and the structural model, and concludes with a discussion of the findings, their theoretical and practical implications, the limitations of the design, and the avenues for future research it opens.

### 1.1. Theoretical Framework and Hypothesis Development

#### 1.1.1. Social Cognitive Theory as an Integrative Framework

Research on artificial intelligence in higher education has largely been built on models of technology acceptance, which effectively explain why a student adopts a tool and how intensively they use it, but say nothing about the normative quality of that use. A student may adopt a generative tool with high behavioral intention and use it to avoid the intellectual work required by the task. Explaining responsible use requires a framework that distinguishes between possessing a capacity and exercising it, and that framework is social cognitive theory.

Bandura (1986) argues that behavior, personal factors, and the environment are mutually determined, and that knowledge does not automatically translate into action. Operating between these two is self-regulatory agency, a system comprising three subfunctions: self-observation of behavior, evaluation of that behavior against personal standards, and an adjustment response (Bandura, 1991). Zimmerman (2000, 2002) applied this system to academic learning and organized it into a cycle of anticipation, execution, and self-reflection. The implication is clear: knowing how a generative system works, what its limits are, and what academic integrity requires is a necessary but not sufficient condition for responsible behavior. Students must also activate the processes that translate that knowledge into concrete decisions when faced with a time-bound task.

The framework assigns a role to each construct in the model. Artificial intelligence literacy functions as the personal competence factor in the structured knowledge that enables action. Self-regulated learning functions as the agency mechanism that translates that competence into behavior. The responsible use of generative AI is the behaviour of interest, captured here through self-report rather than direct observation. And technostress functions as the environmental condition that restricts the exercise of agency. Social cognitive theory is the primary framework of this study, and it alone supplies the model’s architecture: it assigns the role of every construct and it generates the central prediction that competence reaches behaviour through agency rather than directly. The two additional perspectives invoked below are auxiliary and each does one job that social cognitive theory does not do. Person–environment fit theory supplies the definition of the moderator, because social cognitive theory names the environment as a determinant without specifying what makes a technological environment adverse. Resource conservation and the demands–resources model supply the mechanism by which an adverse environment should act, namely by making costly acts the first to be cut, which is what predicts that the moderation falls on the second stage rather than on the first. Neither perspective introduces constructs of its own into the model, and neither is used to explain a relationship that social cognitive theory already explains; where accounts overlap, the social-cognitive reading is the one adopted. For this last role, social cognitive theory requires a complement. Resource conservation theory holds that people seek to obtain and protect the resources they value, and that, when faced with the threat of loss, they reduce their investment in costly activities to preserve what they still have (Hobfoll, 1989). The demands-and-resources model adds that environmental demands deplete available energy, while resources replenish (Bakker & Demerouti, 2017); a logic that has been extended to the student context with evidence on burnout and academic engagement (Salmela-Aro & Upadyaya, 2014). It should be noted that the argument does not rely on the limited-resource hypothesis in its original formulation, which has been called into question by successive replication failures, but rather on the mechanistic revision that explains the decline in self-control through shifts in motivation and attention under sustained demand (Inzlicht & Schmeichel, 2012).

#### 1.1.2. Artificial Intelligence Literacy

Artificial intelligence literacy refers to the set of capabilities that enable a person to recognize the technology operating within the applications they use, employ it effectively, critically evaluate its capabilities and limitations, and assess the ethical implications of its use (B. Wang et al., 2023). These four dimensions (awareness, use, evaluation, and ethics) have been consistently identified in recent reviews. A systematic review of 51 empirical studies identified five generative AI literacy competencies that overlap with these almost point by point and concluded that students demonstrate a moderate understanding of the concepts but encounter persistent difficulties in engineering instructions and critically evaluating the generated outputs (Park, 2025). Another exploratory review proposed a three-dimensional framework (technical competence, ethical responsibility, and social awareness) across five progressive cognitive stages (Zainal et al., 2026), and a third review, focused on frameworks available in higher education, found convergence toward interdisciplinary approaches that combine technical knowledge, ethical considerations, and critical thinking (Rojas-Contreras et al., 2025).

Measurement of the construct has advanced in parallel. Tremblay et al. (2025) validated a five-dimensional questionnaire incorporating human agency among 768 students in Quebec, and Hershkovitz et al. (2025) measured generative AI literacy among 1667 students, finding medium and high levels of proficiency and 82% support for the formal teaching of these competencies. A finding from the same body of literature foreshadows the tension addressed in this study: among 91 economics and business students, 92.3% regularly used AI tools, but only 40.0% understood their applications, and barely 19.0% recognized the core risks (Čolakovac et al., 2026). Operational proficiency is advancing faster than ethical understanding.

#### 1.1.3. Responsible Use of Generative Artificial Intelligence

The responsible use of generative AI is defined here as the pattern of academic conduct that respects institutional rules regarding the use of these tools, subjects the content they produce to verification, and transparently discloses their contribution to the submitted work. One point of terminology follows from how the construct is measured. Everything reported in this study is a self-reported practice: no submitted assignment was inspected, no verification trace was recorded, and no disclosure statement was audited. The construct is therefore referred to as self-reported responsible AI-use practices wherever precision matters, and claims about what students do should be read as claims about what students report doing. These three dimensions are drawn from Chan’s (2023) AI policy framework for higher education, which places the prevention of academic misconduct and the requirement to explicitly attribute AI’s contribution, with the same status as a bibliographic reference, at the core of its governance framework, and from UNESCO’s (2023) guidance, which emphasizes that the outputs of these systems cannot be used without prior critical evaluation because the model does not understand the text it generates.

The available evidence in the Peruvian context confirms that all three dimensions are problematic, though to varying degrees. Reina Marín et al. (2025) surveyed 890 students and 162 faculty members from 21 Peruvian institutions and found that 53.5% of students believe that AI systems lack transparency, and 47.5% express concern about data privacy. Garro-Aburto et al. (2026) validated an academic integrity questionnaire among 419 Peruvian university students; the final structure of the questionnaire retained a factor related to normative integrity in the use of AI, which confirms the relevance of that dimension but excludes verification of content and authorship. Larios Soldevilla et al. (2025) documented at a Peruvian business school that students report improvements in citation management and writing clarity and identify ethical use as an explicit concern. The dimension of authorship appears to be the most fragile: Oré Gálvez et al. (2026) found at a public Andean university a persistent discrepancy between the use students acknowledge and the use they report to the institution, with selective disclosure practices shaped by fear of judgment.

#### 1.1.4. Self-Regulated Learning

Self-regulated learning encompasses the processes through which students set goals, select strategies, manage their time, and evaluate their own progress (Zimmerman, 2000), processes that Barnard et al. (2009) operationalized for technology-mediated environments. The relationship between these processes and generative systems allows for two opposing interpretations, both of which are supported by empirical evidence.

The optimistic interpretation is supported by accumulated evidence. A three-level meta-analysis of 95 effect sizes from 28 studies concluded that interventions using artificial intelligence significantly improve self-regulation (J. Wu et al., 2026). At the experimental level, explicit metacognitive support in generative environments enhanced task strategy and self-assessment (Xu et al., 2025), metacognitive instructions delivered via a customized ChatGPT improved problem-solving processes (Joo et al., 2026), and interactive scaffolding based on generative AI outperformed alternatives in skill development (Ngu et al., 2025). In Peru, metacognitive strategies correlate moderately with autonomous learning (rho = 0.594) among university students in Lima, although 56.6% of the sample exhibited deficient levels (Rojas-Ortega et al., 2025), and a heuristic intervention increased self-regulation among 343 students at a national university (Oseda Gago et al., 2020).

The pessimistic view is no less solid and is more specific. Cognitive offloading is the use of external resources to reduce the demand on one’s own processing (Risko & Gilbert, 2016), which becomes problematic when what is offloaded is not execution but regulation. Fan et al. (2025) found that students who worked with ChatGPT produced better outcomes but did not improve their metacognitive processes a pattern they termed “metacognitive laziness.” The same tension appears in the Peruvian evidence: dependence on AI is negatively associated with critical thinking, particularly in inference and evaluation, leading Vereau Amaya et al. (2025) to argue that unmediated use can displace self-regulatory processes in novice students; and the qualitative analysis of 265 interviews described a shift from instrumental efficiency toward cognitive delegation and a decline in critical autonomy (García Castro et al., 2026). What both perspectives share is that the outcome depends on whether the student regulates the use or whether the use replaces regulation.

#### 1.1.5. Technostress and Person–Environment Mismatch

Technostress is conceptualized here based on person–environment fit theory, which locates the tension not in the person or the environment separately but in the mismatch between the two, either because demands exceed the individual’s capabilities or because the resources provided do not meet their needs (Edwards et al., 1998). Applied to technology, this logic led to the identification of a stable set of sources of technostress and their consequences for satisfaction and engagement (Ragu-Nathan et al., 2008). In the educational setting, X. Wang and Li (2019) developed a multidimensional measure of this mismatch, and X. Wang et al. (2020) adapted it to the student context, with subsequent validation among Peruvian college students that retained three factors (Verde-Avalos et al., 2025).

The evidence on technostress and generative artificial intelligence is recent and inconsistent. Yang and Tang (2026) found among 649 Chinese students that techno stressors related to generative AI affect learning performance through self-efficacy, with effects varying depending on the stressor. Acosta-Enriquez et al. (2025b) demonstrated, among 876 pre-service teachers at twelve Peruvian universities, that stress mediates the relationship between academic overload and the use of AI models. An experimental design involving Peruvian instructors reduced digital fatigue by approximately 22% through a brief content-curation workflow using ChatGPT, within the demands-and-resources framework (Cajas Bravo et al., 2025), and among 258 engineering students, perceived risk predicted resistance to use (Vargas-Bejarano et al., 2025). The most informative finding for this study is negative: Marquez-Yauri et al. (2026) found no direct effect of technostress on critical thinking among 340 Peruvian college students (β = −0.06, *p* = 0.50) nor any support for its moderating role in the relationship between self-efficacy in AI and critical thinking. This result narrows down where the effect should not be sought and motivates the stratification of moderators.

### 1.2. Hypothesis Development

#### 1.2.1. Direct Effects

The ethical dimension is an integral part of the construct of artificial intelligence literacy and is not an afterthought: the reference scale includes vigilance against technological abuse and adherence to ethical principles among its indicators (B. Wang et al., 2023), and reviews of frameworks agree on incorporating ethical responsibility as a central component (Park, 2025; Zainal et al., 2026). On the empirical side, attitudes toward artificial intelligence have a direct impact on academic ethics among 406 students at six Peruvian universities (β = 0.33), with additional indirect effects attributable to moderating factors (Espinoza Vidaurre et al., 2025), and recognition of the risks associated with its use distinguishes between student profiles (Čolakovac et al., 2026). A student who knows how to assess the reliability of a generative output possesses the necessary judgment to decide whether to verify it before incorporating it. The two constructs are not thereby collapsed into one. The ethics facet of AI literacy asks what a student knows and is disposed to notice about the technology in general, such as vigilance against misuse and awareness of ethical principles; the responsible-use construct asks what a student reports having done with a particular tool in a particular graded submission. Knowledge and disposition are not conduct, and the distance between possessing a capacity and exercising it is the premise the model exists to test rather than an ambiguity in it. Because the two nonetheless share ethical content, Section 3.8 reports a sensitivity analysis in which the ethics facet is removed from the predictor. On this basis, we propose:

**Hypothesis 1 (H1).** 
*Artificial intelligence literacy positively predicts the responsible use of generative artificial intelligence.*

The anticipation phase of the self-regulatory cycle requires students to anticipate the demands of the task and select appropriate strategies, which is only possible if they understand the environment in which they will be working (Zimmerman, 2000). The meta-analysis by J. Wu et al. (2026) confirms that structured exposure to artificial intelligence tools improves self-regulation, and Peruvian evidence points in the same direction from the competency perspective: self-efficacy in AI learning was the strongest predictor of technology adoption among 482 college students (β = 0.431; Acosta-Enriquez et al., 2025a), and digital competencies correlate strongly with academic self-efficacy among students in Peru’s central jungle region (González-Prida et al., 2024). Those who understand what a generative system can and cannot do are better equipped to set realistic goals and manage their study time. Consequently:

**Hypothesis 2 (H2).** 
*Artificial intelligence literacy positively predicts self-regulated learning.*

Self-observation and judgment against personal standards are precisely the processes required for the academically responsible use of a generative tool (Bandura, 1991). Experimental evidence confirms this in two ways: explicit metacognitive support improved task strategy and self-assessment in generative environments (Xu et al., 2025), and metacognitive instructions aimed at the responsible use of AI reduced learning anxiety and increased academic self-efficacy in a classroom experiment with 148 participants (Jiang & Xu, 2026). Peruvian evidence confirms this by contrast: where self-regulation is absent, AI use leads to dependence and a deterioration in inference and evaluation (Vereau Amaya et al., 2025) or to overt cognitive delegation (García Castro et al., 2026). Therefore, we propose:

**Hypothesis 3 (H3).** 
*Self-regulated learning positively predicts the responsible use of generative artificial intelligence.*

#### 1.2.2. The Mediating Role of Self-Regulated Learning

The central premise of social cognitive theory is that personal factors influence behavior through agency-based processes rather than directly and comprehensively (Bandura, 1986, 1991). Applied to the problem at hand, this premise implies that proficiency in artificial intelligence should lead to responsible use to the extent that it activates processes of goal setting, strategy selection, time management, and self-evaluation. The finding regarding metacognitive laziness reinforces this interpretation from a negative perspective, showing that product improvement can occur without any improvement in the regulatory process (Fan et al., 2025). Peruvian literature has mentioned this mechanism on two occasions without modeling it: Vereau Amaya et al. (2025) describe it as a displacement of self-regulatory processes, and García Castro et al. (2026) as a reduction in critical autonomy. On this basis, we propose:

**Hypothesis 4 (H4).** 
*Self-regulated learning positively mediates the relationship between artificial intelligence literacy and the responsible use of generative artificial intelligence.*

#### 1.2.3. Technostress as a Moderating Condition

The following three hypotheses specify where technostress intervenes within the mediated process. Resource conservation theory predicts that, when faced with the threat of loss, people cut back on investment in costly activities to protect what they have (Hobfoll, 1989), and the mechanistic view of self-control holds that under sustained demand, regulatory effort declines due to shifts in motivation and attention (Inzlicht & Schmeichel, 2012). The critical point is that the three pathways of the model do not impose the same burden.

Acquiring and applying technical competence is an activity that technostress could make more difficult, as the mismatch between technological demands and available capabilities reduces the willingness to explore the tool (Edwards et al., 1998). Generative AI techno stressors impair learning performance through the self-efficacy pathway (Yang & Tang, 2026), and perceived risk predicts resistance to using these tools (Vargas-Bejarano et al., 2025). It should be noted, however, that the only Peruvian study examining a similar moderation effect found no support for it (Marquez-Yauri et al., 2026); thus, the hypothesis is proposed based on theoretical expectations but also with an empirical caveat:

**Hypothesis 5 (H5).** 
*Technostress negatively moderates the relationship between artificial intelligence literacy and self-regulated learning, such that the relationship weakens as technostress increases.*

The second stage imposes a different burden. Verifying a generative output against independent sources and documenting the tool’s contribution to a deliverable are deliberate, time-consuming acts carried out under tight deadlines exactly the kind of investment that the resource conservation logic predicts will be cut first when stress increases (Bakker & Demerouti, 2017; Hobfoll, 1989). The evidence regarding students is consistent: digital fatigue and overload are associated with usage patterns aimed at immediate relief from demand (Acosta-Enriquez et al., 2025b; Cajas Bravo et al., 2025), and AI-related learning anxiety reduces academic self-efficacy, which is the resource that sustains regulatory effort (Jiang & Xu, 2026). Consequently:

**Hypothesis 6 (H6).** 
*Technostress negatively moderates the relationship between self-regulated learning and the responsible use of generative artificial intelligence, such that the relationship weakens as technostress increases.*

The direct path captures the effect of competence that does not involve self-regulation; that is, rule-following operates through knowledge of the rule rather than strategic deliberation. If technostress reduces the capacity to sustain deliberate behavior, it should also attenuate this pathway (Inzlicht & Schmeichel, 2012), although the absence of a direct effect of technostress on cognitive outcomes in the Peruvian population (Marquez-Yauri et al., 2026) suggests that the attenuation might be less than in the previous pathways. On this basis, we propose:

**Hypothesis 7 (H7).** 
*Technostress negatively moderates the direct relationship between artificial intelligence literacy and the responsible use of generative artificial intelligence, such that the relationship weakens as technostress increases.*

#### 1.2.4. Moderated Mediation

When a moderator acts on any of the stages of a mediated process, the magnitude of the indirect effect is no longer constant and becomes dependent on the level of the moderator, which requires explicitly testing for moderated mediation rather than inferring it from the significance of the interaction terms separately (Hayes, 2022). The theoretical argument developed in the previous hypotheses leads to this prediction: if technostress makes any of the stages through which competence is translated into behavior more difficult, the indirect effect of AI literacy on responsible use should be greater among students with low technostress and lower among those who experience it intensely. None of the reviewed studies have tested this prediction. Therefore, we propose:

**Hypothesis 8 (H8).** 
*The indirect effect of artificial intelligence literacy on the responsible use of generative artificial intelligence, mediated by self-regulated learning, is conditional on the level of technostress and decreases as technostress increases.*

### 1.3. Research Model

**Figure 1:**
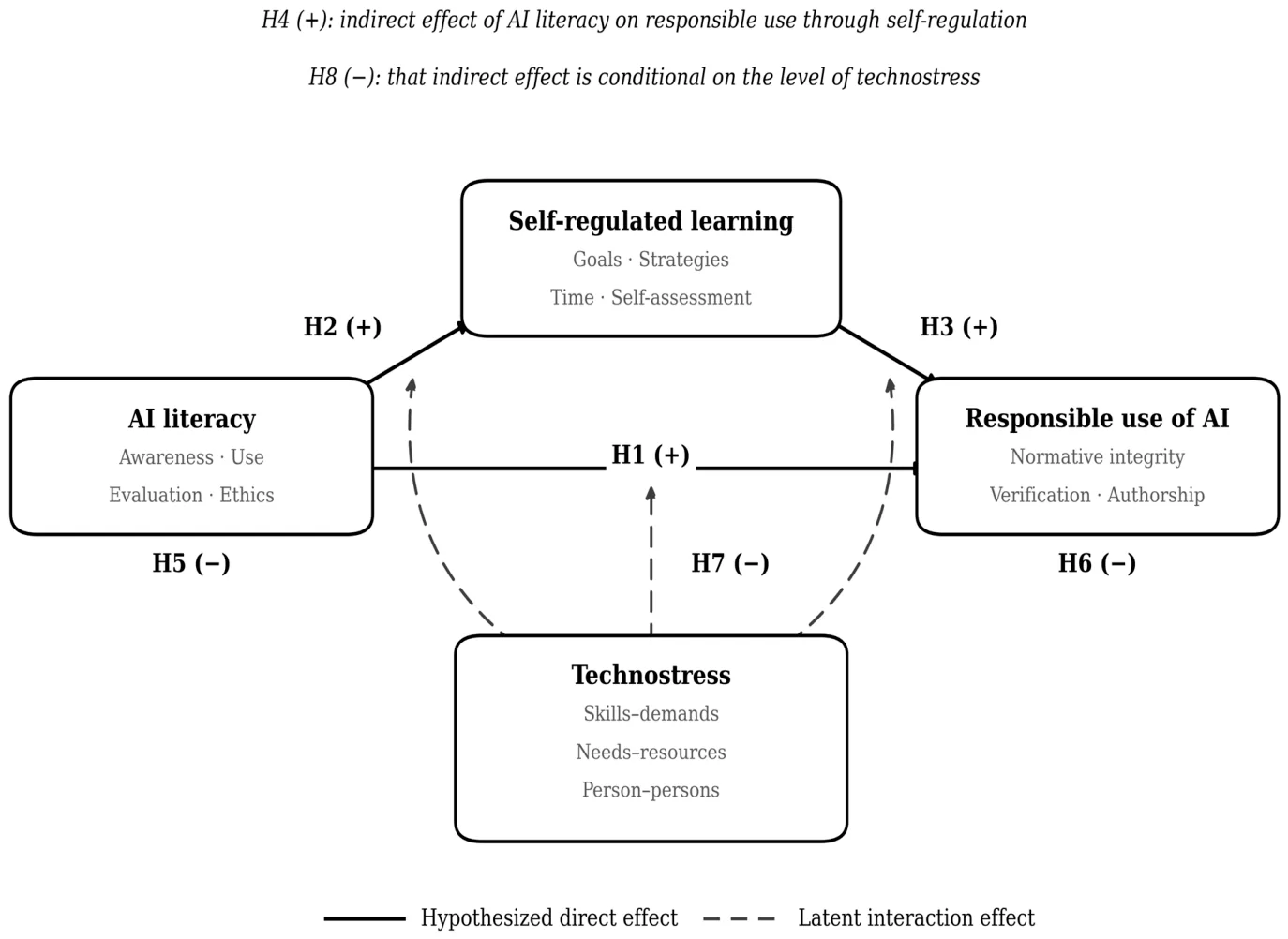
Proposed research model. *Note.* The solid lines represent the hypothesized direct effects, and the dashed lines represent the interaction effects. H4 refers to the indirect effect of artificial intelligence literacy on responsible use through self-regulated learning, and H8 refers to the conditionality of that indirect effect on the level of technostress.

Table 1 summarizes the empirical evidence supporting each segment of the model and highlights the two relationships that the literature has not tested. Figure 1 depicts the complete network: three direct effects, one indirect effect, and three latent interaction terms that condition the first stage, the second stage, and the direct path.

Figure 1 translates this evidence into the model to be tested. It brings together the four constructs, the path that runs from literacy to responsible use through self-regulated learning, and the two points at which technostress is expected to alter the strength of that sequence.

## 2. Materials and Methods

### 2.1. Research Design

The study adopted a quantitative approach with a non-experimental, cross-sectional design aimed at explanatory analysis. The research question driving this study is not descriptive. The aim was not to determine the level of artificial intelligence literacy among Peruvian students, but rather to estimate the magnitude and direction of a hypothesised mechanism linking that literacy to the responsible use of generative AI through self-regulated learning, and to determine at which levels of technostress that association is weaker. A question formulated in terms of process and conditions requires a model that estimates several equations simultaneously and separates true variance from measurement error. Because the data were collected at a single point in time, every coefficient reported below is an association consistent with the hypothesised process, not an estimate of a causal effect. Mediation and moderation are used here as statistical specifications, and the terms mediator, indirect effect and conditional effect carry no temporal or causal claim; the design-based limits of cross-sectional mediation are set out in Section 4.7. A series of independent regressions does not achieve this.

The eight hypotheses were therefore tested using covariance-based structural equation modeling, employing the two-stage strategy of Anderson and Gerbing (1988): first, the measurement model was evaluated, and only afterward once the psychometric quality of the four constructs had been established was the structural model estimated. The covariance-based approach was preferred over the variance-based approach for three reasons. The objective was to test a pre-formulated theory rather than to maximize the prediction of the dependent variable. The four constructs are reflective and have previously validated scales. Furthermore, only covariance-based estimation provides absolute fit indices and formal tests of measurement invariance (Bollen, 1989; Kline, 2023). The structural specification corresponds to a two-stage moderated mediation model with additional moderation of the direct path that is, the latent analog of Hayes’s (2022) Model 59 with the difference that here the four variables were modeled as latent variables rather than observed scores.

### 2.2. Population, Sample, and Sampling

The target population consisted of undergraduate students currently enrolled in Peruvian universities. The latest available official figure estimates this population at around 1.3 million people, with 66% in private institutions and 34% in public ones (National Superintendency of Higher Education, 2021). Three eligibility criteria were established: at least 18 years of age, a requirement verified on the consent form as a prerequisite for accessing the questionnaire; current enrollment in an undergraduate program during the survey period; and prior experience with at least one generative AI tool at any point before the survey, verified through a dichotomous screening question at the beginning of the questionnaire, which was the sole eligibility filter applied to use. This screening item and the frequency item reported in Table 2 refer to different periods and are not interchangeable: the screening item asks whether the student has ever used a generative AI tool, whereas the frequency item asks how often the student used such tools for academic work during the survey period. The two are therefore compatible, and the 81 respondents (9.9%) who reported no academic use during the survey period had all confirmed prior experience at screening; they are students who had used these tools before but did not use them for coursework in the weeks covered by the questionnaire. Cases with incomplete responses were excluded.

Sampling was non-probabilistic, based on convenience with chain propagation. This decision was driven by the absence of an accessible sampling framework: Peru lacks a public, up-to-date registry of undergraduate students that would allow for a random draw on a national scale, and institutional directories are protected by personal data regulations. Rather than presenting this as an unavoidable shortcoming, it is important to clarify what kind of inferences it enables. Jager et al. (2017) show that a convenience sample ceases to be a questionable shortcut when the specific population it represents is precisely defined and its composition is documented, because controlled homogeneity enhances the internal validity of the theoretical test. That is the purpose here: to test a mechanism, not to estimate population parameters. The limitation on generalizability is also explicitly acknowledged (Andrade, 2021) and is addressed in the section on limitations. One consequence of this recruitment strategy should be stated at the outset. Students reached through classrooms and through institutional networks may be clustered within universities, courses and referral chains, and observations within such clusters are not strictly independent, so standard errors estimated under an independence assumption may be too small. The anonymisation protocol described in Section 2.4 did not retain institutional, faculty or classroom identifiers, so the number of recruitment clusters is not recoverable from the analytic matrix and cluster-robust or multilevel estimation was not possible. Section 3.8 reports what the stratification variables that were retained allow on this point, and Section 4.7 treats the matter as a limitation of the design.

To counteract excessive homogeneity, deliberate variation was sought in the dimensions identified as relevant in the literature. The sample was distributed across public and private universities, between Metropolitan Lima and other regions, across the five major fields of study, and across the ten academic cycles. Table 2 documents the results.

The final sample consisted of 820 students. Its adequacy was justified through four converging approaches rather than relying on a single rule.

The first is the priori statistical power for the most demanding test in the model, which is not a main effect but rather the latent interaction term. An analysis using G*Power 3.1 (Faul et al., 2009) for the increase in R^2^ associated with an additional predictor in a model with five predictors with a small effect size (f^2^ = 0.02), α = 0.05, and power of 0.80 indicated a requirement of 395 cases. The second is the sensitivity analysis, which is more informative than a priori power because it is based on the actual sample size obtained: with 820 cases, α = 0.05, and at a power of 0.80, the study detects interaction effects as small as f^2^ = 0.0096, half the conventional threshold for a small effect (Cohen, 1988). The third is power based on RMSEA for the non-adjacent fit contrast (H_0_: RMSEA = 0.05 versus H_1_: RMSEA = 0.08) according to the procedure by MacCallum et al. (1996): for the aggregate measure model, with 71 degrees of freedom, 167 cases would suffice to achieve a power of 0.80, so that with 820 cases, the power is practically complete.

The fourth measure is the ratio of cases to free parameters, which in the main structural model which, with 51 parameters, reaches 16.1 to 1, exceeding the minimum of 10 to 1 and approaching the ideal ratio of 20 to 1 recommended by Kline (2023). The ratio of cases per item is 17.4 to 1. Wolf et al. (2013) demonstrate through simulation that models with mediation and multiple indicators per factor require between 300 and 500 cases for unbiased estimates and eigenvalues; the 820 available cases comfortably exceed that range.

### 2.3. Measurement Instrument

The questionnaire was organized into three sections. The first section presented information about the study, the informed consent form, and the eligibility screening question. The second section collected the eleven sociodemographic and context-of-use variables listed in Table 2, including the eight that later served as control covariates. The third block contained 47 items across the four constructs, presented in separate blocks by scale, with the order of items randomized within each block. All four constructs, the outcome included, are self-report measures obtained from the same respondents in a single administration. All items were rated on a five-point Likert scale, ranging from 1 (*strongly disagree*) to 5 (*strongly agree*).

Artificial intelligence literacy was measured using the twelve items from the *Artificial Intelligence Literacy Scale* by B. Wang et al. (2023), which allocates three items to each of its four dimensions: awareness, use, evaluation, and ethics. The original scale uses a seven-point response format. Here it was reduced to five points for two reasons. The instrument as a whole contains 47 items drawn from four sources, and holding the response format constant across blocks prevents the format changes from becoming a source of systematic variance in their own right, a procedural safeguard against common-method bias (Podsakoff et al., 2003). The remaining three blocks were five-point scales in the versions validated for this population, so standardising downward preserved those validations rather than altering three instruments to accommodate one. The trade-off is explicit: reducing the number of categories can slightly lower observed variance and reliability, although the loss is small from seven to five points and negligible beyond five categories (Lozano et al., 2008; Simms et al., 2019), and the reliability obtained here (α = 0.937 for the total scale) matches that reported for the seven-point original. The cost is comparability: mean scores on this five-point version are not directly comparable with published seven-point scores, and only the correlational and structural evidence transfers across versions. This is noted again in Section 4.7. The two items that were reverse scored in the original version were recoded prior to analysis.

Self-regulated learning was measured using twelve items adapted from *the Online Self-Regulated Learning Questionnaire* by Barnard et al. (2009), with Zimmerman’s (2002) three-phase cyclical model as the conceptual framework and the Spanish version validated by Pinto Santubera et al. (2020) as the linguistic framework. The original questionnaire contains 24 items across six subscales. Four of these were retained, with three items each: goal setting, learning strategies, time management, and self-assessment. The two discarded subscales, environmental structuring and help-seeking, relate to the regulation of the physical and social conditions of study, whereas the theoretical rationale of this study concerns the regulation that students themselves exert over their cognitive process when they delegate tasks to a generative system. The reduction was therefore based on conceptual relevance and decided a priori, before any data were examined, rather than by discarding subscales that performed poorly. Its consequences were then tested empirically: the four retained subscales reproduced their intended structure with standardised loadings between 0.758 and 0.855 and reliabilities between 0.806 and 0.836, and the second-order factor they define fits as well as the model with fourteen freely correlated dimensions (Section 3.2). Two qualifications follow. Removing environmental structuring and help-seeking narrows the construct, so what is measured here is the regulation students exert over their own cognitive process and not self-regulated learning in the full breadth of the original questionnaire; the label should be read accordingly. And the resulting twelve-item version is not score-comparable with the 24-item OSLQ, so the coefficients reported here should be compared with the published literature at the level of relationships between constructs, not at the level of scale means. This is noted again in Section 4.7.

Technostress was measured using eleven items organized into three dimensions based on the type of person–environment mismatch captured by each block: mismatch between skills and demands (four items), mismatch between needs and resources (four items), and mismatch between the individual and others (three items). The source instrument is the scale by X. Wang and Li (2019), developed based on the multidimensional theory of person–environment mismatch, along with its adaptation to the student context by X. Wang et al. (2020). The version used draws on the Peruvian validation by Verde-Avalos et al. (2025), which tested 22-, 20-, and 19-item versions and retained an 11-item structure across three factors with the same distribution of four, four, and three items as used here. This background is relevant for a specific reason: successive adaptations in Spain (Penado Abilleira et al., 2020) and Chile (Vega-Muñoz et al., 2022) show that the original factor structure does not hold up without adjustment in Spanish-speaking contexts. Starting with a version already calibrated for the Peruvian university population reduces that risk.

At the time of the study’s design, there was no validated scale for the responsible use of generative AI; therefore, a custom instrument was developed consisting of twelve items across three dimensions, each with four items: regulatory integrity, content verification, and authorship and transparency. The three dimensions were derived from Chan’s (2023) AI policy framework for higher education, which places both the prevention of academic misconduct and the requirement to explicitly attribute AI’s contribution at the core of its governance framework, and from UNESCO’s (2023) guidance, which emphasizes that the outputs of generative systems cannot be used without prior critical evaluation. The three dimensions are not an arbitrary partition. Chan’s (2023) framework separates what a student must not do (breach the institution’s rules) from what a student must do (attribute the tool’s contribution), and UNESCO’s (2023) guidance adds a third obligation that neither of those covers, namely the critical appraisal of an output the model does not itself understand. Normative integrity, authorship and transparency, and content verification correspond one to one with those three obligations, and they differ in the cost they impose on the student: complying with a known rule is a low-cost act, whereas verification and disclosure are deliberate and time-consuming. That difference in cost is what makes the three-dimensional structure theoretically consequential for this study rather than merely descriptive. The items were drafted using the functional procedure outlined by Haynes et al. (1995): prior definition of the domain in behavioural terms for each dimension, generation of a deliberately over-inclusive initial pool of items for each dimension, and refinement through expert judgment, after which the pool was reduced to the twelve items retained. Items were eliminated when they were double-barrelled, when they duplicated the content of a retained item, or when they fell below the quantitative retention criterion specified below. The normative integrity dimension aligns with the factor of the same name that Garro-Aburto et al. (2026) subsequently validated among 419 Peruvian university students, providing independent support for the construct’s relevance in this context. The normative integrity items ask whether the student adheres to the rules they understand to govern their coursework, not whether they can recall a written institutional policy. The two are distinct questions, and the first remains answerable where no policy exists or where the student does not know whether one does, since expectations about acceptable use are conveyed by instructors and course documents as well as by formal regulation. Because a reader may still doubt this, Section 3.8 reports a sensitivity analysis by policy awareness.

The three adapted instruments were translated from English into Spanish using Brislin’s (1970) translation and back-translation procedure, following the five-stage sequence systematized by Beaton et al. (2000) and in accordance with the guidelines of the International Test Commission (2018). Two independent bilingual translators produced Spanish versions; a committee consolidated them into a draft version; two different translators, who were not involved in the original translations, back translated it into English; and the committee compared both versions to resolve semantic and conceptual equivalence discrepancies.

The content validity of the resulting version, including the self-developed items, was examined through expert judgment by five judges specializing in educational technology, psychometrics, and research ethics. Each judge rated the adequacy, clarity, coherence, and relevance of each item. L. R. Aiken’s (1985) was calculated along with its 95% confidence interval, as well as the content validity index per item and per scale (Lawshe, 1975; Polit & Beck, 2006), using the application protocol developed by Escobar-Pérez and Cuervo-Martínez (2008). Items with a V ≥ 0.80 and a lower limit of the interval greater than 0.70 were retained; the set of items achieved an average V of 0.87, and the twelve items of the responsible-use scale, which had no prior published validation, met the same criterion. Content validation and satisfactory internal structure are necessary but not sufficient conditions for a new measure: the evidence reported here establishes that the responsible-use items behave as three coherent and reliable facets in this sample, and it does not establish that the scale is generalisable. Independent validation in samples not involved in its construction, including confirmation of its factor structure and evidence of convergent and criterion validity against external indicators, remains necessary before the scores are used comparatively; this is stated again among the limitations in Section 4.7. A further constraint should be stated plainly: the scale was developed, refined by expert judgment, piloted, psychometrically examined and then used for substantive hypothesis testing within the same programme of work, and its psychometric evaluation and the structural models reported below were carried out on the same 820 cases. The two sets of evidence are therefore not independent of one another. What is offered here is preliminary psychometric support for a new instrument, not definitive validation of it. The refined version was administered in a pilot test to 80 students with characteristics equivalent to those of the target sample who were not included in the final analysis, to verify comprehension of the items, estimate response time, and obtain preliminary reliability by scale, which proved satisfactory (α = 0.82). Table 3 summarizes the operationalization of the four constructs, with their dimensions, codes, number of items and anchor items.

### 2.4. Data Collection Procedure and Ethical Considerations

Data collection was conducted between May and June 2026 using a mixed-mode administration procedure. The term refers to the two channels through which the same quantitative questionnaire was delivered and not to a mixed-methods design: no qualitative data were collected, and the study is quantitative throughout. A portion of the questionnaires was administered in person and under supervision in classrooms at the participating institutions, following prior coordination with the responsible instructors. The remainder was administered via an online form distributed through institutional email, virtual classrooms, and student groups, using a chain-mailing approach. The combination of both channels expanded geographic coverage without sacrificing control over the administration conditions for a portion of the sample. Both channels delivered an identical instrument with identical item order randomisation. The online form prevented submission with unanswered items and allowed only one response per device, which explains why the final matrix contains no missing values. Mode of administration was not retained as a case-level variable in the anonymised matrix. It could therefore not be entered as a covariate, nor tested for measurement invariance, and the possibility that supervised and unsupervised administration elicited different levels of reporting on socially sensitive items cannot be ruled out. This is treated as a limitation in Section 4.7. The average response time was 10 min. The 820 cases analysed here were collected specifically for this study during that period and constitute an independent sample: they do not overlap with the participants of previously published studies by members of this author team, including Marquez-Yauri et al. (2026).

All participants read an information sheet explaining the study’s purpose, the intended use of the data, the voluntary nature of participation, and the option to withdraw at any time without any consequences, and they provided their informed consent before accessing the questionnaire. No financial or academic incentives were offered. No data that could identify the participants or their institutions were collected, and the matrix was analyzed anonymously and in aggregate form. The study was conducted in accordance with the principles of the Declaration of Helsinki (World Medical Association, 2025) and the National Code of Scientific Integrity in force in Peru (National Council for Science, Technology, and Technological Innovation [Concytec], 2024) and was approved by the Ethics Committee 2026-IIICyT-ITCA under approval code 0178-2026-GM-IIICyT, dated 18 April 2026.

### 2.5. Data Analysis Strategy

The analysis was performed using Mplus 8 (L. K. Muthén & Muthén, 1998–2017), and power calculations were conducted using G*Power 3.1 (Faul et al., 2009). The procedure was organized into two phases; this division is not for presentation purposes but rather due to an estimator restriction that should be noted before describing the steps. The XWITH operator the only native Mplus procedure for estimating interactions between latent variables requires numerical integration and maximum likelihood estimation and is not available under WLSMV. Furthermore, WLSMV based on polychoric correlations is the appropriate estimator for five-category ordinal items and the only one that produces absolute fit indices and defensible tests of measurement invariance (B. Muthén, 1984; Rhemtulla et al., 2012; Li, 2016). Rather than sacrificing one of the two requirements, each question was assigned its corresponding estimator.

#### 2.5.1. Measurement Phase

The 47 items were treated as ordinal and estimated using WLSMV on polychoric correlations with delta parameterization. Four specifications were compared: the first-order model with the fourteen correlated dimensions, the second-order model that nests those dimensions under the four theoretical constructs, a rival four-factor model at the item level, and a unifactorial model. Model fit was assessed using CFI and TLI ≥ 0.95 as acceptable criteria and ≥0.90 as a minimum, RMSEA ≤ 0.06 and ≤0.08, respectively, and SRMR ≤ 0.08 (Hu & Bentler, 1999), interpreted with the caution recommended by Marsh et al. (2004) regarding rigid cutoff points and with the caveat that the indices are not directly comparable across estimators (Xia & Yang, 2019). Standardized loadings of ≥0.70 were required, with 0.50 as the minimum acceptable value (Hair et al., 2019a).

Reliability was estimated using Cronbach’s alpha (Cronbach, 1951), McDonald’s omega (McDonald, 1999), and Jöreskog’s composite reliability (Jöreskog, 1971), with a threshold of 0.70. Convergent validity was assessed using the average variance extracted, with a threshold of 0.50 (Fornell & Larcker, 1981). For discriminant validity, two complementary criteria were used: the Fornell–Larcker criterion, which requires that the square root of the average extracted variance of each construct exceed its correlation with any other construct, and the heterotrait–monotrait ratio with a strict threshold of 0.85 (Henseler et al., 2015), which Voorhees et al. (2016) show to be more sensitive than the former. Composite reliability and mean extracted variance were derived from the fully standardized loadings of the WLSMV model rather than from the alpha coefficient on raw scores, because this approach accounts for the ordinal nature of the items and does not assume equal loadings across indicators.

Measurement invariance was tested by gender and by type of university using the four-factor model at the item level, through the configural, metric, and scalar sequence with theta parameterization, which corresponds to categorical indicators (Vandenberg & Lance, 2000). The ten cases that did not report gender were excluded from the first comparison. Since, with samples of this size, the chi-square difference test rejects discrepancies without substantive consequences, the decision was based on fit indices, with thresholds of ΔCFI ≤ 0.010 and ΔRMSEA ≤ 0.015 (Cheung & Rensvold, 2002; Chen, 2007; Meade et al., 2008).

Common-method bias was first addressed procedurally, in the instrument’s design itself: visual separation of scale blocks, randomization of item order within each block, guaranteed anonymity, and omission of items with evident social desirability (Podsakoff et al., 2003, 2012). Statistical control was performed through three redundant approaches: the unifactorial model as a confirmatory version of the single-factor test, an unmeasured latent method factor loading on the 47 items, and its equivalent loading on the fourteen dimension scores according to the procedure of Liang et al. (2007). The threshold for concern was set such that the method factor explained more than 25% of the variance (Fuller et al., 2016).

#### 2.5.2. Structural Phase

The structural phase was estimated based on fourteen dimension scores, calculated as the average of the items in each theoretical dimension. This domain-based rather than random aggregation makes it possible to perform the numerical integration required by latent interaction and is justified because the preliminary phase documents that each dimension is unidimensional and reliable. Order matters: without the measurement phase, aggregation would be an arbitrary decision. Measurement error is not disregarded by this step, because the fourteen dimension scores enter the structural model as indicators of the four latent constructs and not as the constructs themselves. Each score carries a freely estimated loading and a freely estimated residual variance, so the portion of its variance that is not shared with the other indicators of its construct is removed from the structural estimates. In these data, that portion ranges from 21.4% to 36.4% per score, with a mean of 27.2%, and the structural coefficients are therefore estimated on the disattenuated latent covariances. What the aggregation forgoes is not the correction for measurement error but the item-level modelling of it, which the two phases separate deliberately: the ordinal item-level structure and its reliability are established under WLSMV in the measurement phase, and the aggregated indicators carry that established structure into the phase where numerical integration is required. The same reasoning governs the latent interaction, which is formed between the latent variables and not between the observed composites, so the product term is disattenuated as well. The gain is quantifiable. Estimating the identical specification on the observed composite scores rather than on the latent variables yields a second-stage interaction of −0.182 against the −0.211 obtained here, an attenuation of 13.8%, with corresponding attenuations of 8.7% and 7.3% for the two principal structural paths; the uncorrected estimates are biased towards zero, as measurement-error theory predicts, and the latent specification removes that bias.

Three models were estimated. The first verified, using robust maximum likelihood, that the four-construct structure is replicated in the aggregated scores, and this is the source of the absolute fit indices attributed to the structural model. The second model estimated simple mediation without interaction terms, using maximum likelihood and 10,000 bootstrap resamples with bias correction, which is the standard benchmark for the indirect effect (Preacher & Hayes, 2008; MacKinnon, 2008; Hayes, 2022); this analysis yielded the fully standardized coefficients for H1 through H3 and the one-sided confidence interval for the indirect effect of H4. Mediation was characterized based on the joint significance of the indirect and direct effects, rather than using the stepwise procedure proposed by Baron and Kenny (1986), whose lower power for detecting indirect effects is well documented. Following the cautionary advice of Rucker et al. (2011), the distinction between partial and full mediation is used to describe the distribution of the effect and not as a decision criterion, which is based on the bootstrap interval of the indirect effect.

The third is the main model. It introduces the two latent interaction terms, AIL × TSS and SRL × TSS, using the XWITH operator, which implements the moderated latent structural equation method of Klein and Moosbrugger (2000), with 5000-point Monte Carlo integration and following the application protocol of Maslowsky et al. (2015) and Cheung et al. (2021). The latent variance of the two exogenous variables was fixed at 1, with all their loadings set to free, so that the coefficients are expressed in units of latent standard deviation, and the conditional effects evaluated at −1, 0, and +1 are interpreted directly as one standard deviation below the mean, the mean, and one standard deviation above the mean (L. S. Aiken & West, 1991; Dawson, 2014). The mediator and the dependent variable retained the marker-indicator scaling, because fixing the variance of an endogenous variable would fix the residual variance rather than the total variance.

Since numerical integration does not produce absolute fit indices, the contribution of the interaction terms was assessed using the scaled log-likelihood difference test (Satorra & Bentler, 2010) and by comparing the AIC and BIC with those of the model without interactions (Béland et al., 2022). The indirect, direct, and conditional total effects were calculated at the three conventional levels of the moderator and on a sixteen-point grid between −2 and +2 standard deviations; the moderated mediation contrast (Δ ind) is the difference between the indirect effect at the extreme levels of the moderator. The region of significance was determined using the Johnson–Neyman procedure (Preacher et al., 2006), reconstructed from the parameter covariance matrix to allow for finer gridding than that of the standard output. The confidence intervals for the conditional effects are symmetric and derived from the delta method, because bootstrap resampling is not compatible with numerical integration. This limitation is noted in the corresponding table footnote and is addressed by also reporting the unconditional indirect effect with a bias-corrected bootstrap interval.

Robustness was examined using two additional specifications, both with 2000-point Monte Carlo integration, which incorporates the following covariates: gender, age, academic cycle, type of university, employment status, frequency of generative AI use, training received, and the existence of an institutional policy. The categorical covariates were recoded into dummy variables, with male gender, public university, full-time student status, and the absence of an institutional policy as reference categories. The covariates were selected because each is a plausible common cause of the exogenous variables and the outcome, not because a larger set of controls makes a model more defensible. Gender, age, academic cycle, university type and employment status index the student’s position in the system and were included on that basis. Frequency of generative AI use, training received and the existence of an institutional policy require a caveat, since each may be a consequence of AI literacy rather than an antecedent of it; conditioning on a variable that lies downstream of the predictor removes part of the association of interest and can bias an estimate rather than purge it. They are retained because omitting them would leave an obvious alternative account unexamined, but the fully adjusted specification is reported as a conservative bound obtained under a deliberately, and possibly excessively, adjusted set of controls. It is not the preferred model, and the unadjusted specification remains the model of record. In the first specification, the covariates predict the mediator and the dependent variable. In the second, more heavily adjusted specification, they also predict the two exogenous variables, thereby defining the latent interaction in terms of the components of AI literacy and technostress that are independent of the sample’s sociodemographic composition; as both become endogenous, fixing them at 1 affects their residual variance, and the moderator is measured in terms of residual standard deviations. The magnitude of the effects was interpreted using Cohen’s (1992) guidelines and with the caveat provided by Funder and Ozer (2019) regarding their literal interpretation in applied research.

All decision criteria were established before examining the results. Table S1 in the Supplementary Material summarizes them, and Table 4 associates each hypothesis with the test that contrasts it.

## 3. Results

### 3.1. Preliminary Analysis

The data matrix was complete: all 820 cases responded to the 47 items with no missing values, and all responses fell within the range of scale. The univariate distribution remained within limits that do not compromise the estimation, with skewness between −0.41 and 0.32 and kurtosis between −0.73 and −0.44, values well below the thresholds of |skewness| < 2 and |kurtosis| < 7, beyond which the estimation is compromised (Kline, 2023), and no response category was empty or sparsely populated: the least frequent cell contained 12 cases, clearly above the minimum required for a stable estimation of polychoric correlations (B. Muthén, 1984; Olsson, 1979). The sample of 820 cases comfortably exceeds the recommended minimums for models with robust estimation and a high number of parameters (Kline, 2023) and the sample size derived from the a priori power calculation (Faul et al., 2009). The mean scores paint a picture consistent with the literature on the adoption of generative AI in higher education: AI literacy falls above the midpoint of the scale (*M* = 3.46, *SD* = 0.82), as do responsible use (*M* = 3.45, *SD* = 0.80) and self-regulated learning (*M* = 3.26, *SD* = 0.84), while technostress falls below the midpoint (*M* = 2.77, *SD* = 0.83). The descriptive statistics and factor loadings for the 47 items are presented in Table S2 in the Supplementary Material.

### 3.2. Measurement Model

Following the two-stage strategy of Anderson and Gerbing (1988), the measurement model was evaluated and refined before estimating any structural paths, and all models were estimated in Mplus 8 (L. K. Muthén & Muthén, 1998–2017). The first-order model with the 14 correlated dimensions fit the data excellently, χ^2^ (943) = 896.61, *p* = 0.858, CFI = 1.000, TLI = 1.001, RMSEA = 0.000, 90% CI [0.000, 0.006], SRMR = 0.018; the values comfortably meet the criteria of CFI and TLI ≥ 0.95, RMSEA ≤ 0.06, and SRMR ≤ 0.08 (Hu & Bentler, 1999), interpreted collectively rather than as rigid thresholds (Marsh et al., 2004), and the RMSEA range further supports the close-fit hypothesis (Browne & Cudeck, 1993). The second-order model, which nests these dimensions under the four theoretical constructs and serves as the reference measurement model, yielded an equivalent fit: χ^2^ (1014) = 928.65, *p* = 0.974, CFI = 1.000, TLI = 1.002, RMSEA = 0.000, SRMR = 0.020. The fact that imposing the second-order structure does not impair the fit supports the interpretation of the four constructs as entities with distinct facets rather than as undifferentiated aggregates of items. One caution attaches to the aggregated model reported in the final row of Table 5. Its indices describe the fit of a fourteen-indicator model and are not a second confirmation of the 47-item structure, since aggregation can conceal misspecification at the item level by averaging it away. Item-level fit is established by the models above it in the same table and should be read there; no aggregated model is offered as evidence about item-level structure.

The two rival models confirmed this interpretation (Table 5). The unifactorial model, the confirmatory version of Harman’s test, deteriorated drastically, CFI = 0.549, TLI = 0.529, RMSEA = 0.146, SRMR = 0.151, arguing against the possibility that a single general factor explains the observed covariance. The four-first order-factor model at the item level showed reasonable fit, CFI = 0.991, RMSEA = 0.021, SRMR = 0.026, although it was inferior to the second-order model, indicating that the intermediate dimensional structure provides information and is not a superfluous refinement. No post hoc modifications guided by modification indices were introduced, given the risk of capitalizing on chance and compromising the model’s replicability (MacCallum et al., 1992), and the specification was maintained as derived from the theoretical foundations of the construct (Bollen, 1989; Brown, 2015).

The standardized loadings of the items on their dimensions ranged from 0.758 to 0.860 (*M* = 0.815), meaning that all exceed by a wide margin the 0.70 threshold recommended for reflective indicators (Hair et al., 2019a, 2019b). The loadings of the dimensions on the second-order constructs ranged from 0.893 to 0.959, confirming that the facets share a substantial common core within each construct (Table 6).

Reliability was high at all three levels of aggregation. The 14 dimensions achieved composite reliabilities ranging from 0.836 to 0.894 and average variances extracted ranging from 0.630 to 0.704. McDonald’s omega, announced in the analysis plan, is reported here alongside the other two coefficients: across the fourteen dimensions, it ranged from 0.807 to 0.870, and at construct level it reached 0.937 for AI literacy, 0.937 for self-regulated learning, 0.928 for technostress and 0.935 for responsible use. The three coefficients agree to within 0.002 at every level of aggregation, which is what homogeneous loadings produce and which confirms that no conclusion depends on the choice of reliability estimator. At the construct level, composite reliability ranged from 0.933 to 0.946, and average variance extracted ranged from 0.561 to 0.592. All indicators exceed the conventional thresholds of CR ≥ 0.70 and AVE ≥ 0.50 (Bagozzi & Yi, 1988; Fornell & Larcker, 1981; Hair et al., 2019b), thus establishing convergent validity. Composite reliability is reported alongside alpha because the latter assumes tau-equivalence and underestimates internal consistency in congenic scales such as those used here (Cronbach, 1951; Jöreskog, 1971; McDonald, 1999; Sijtsma, 2009); the convergence between the two coefficients, and their distance from the minimum threshold of 0.70 set for basic research (Nunnally & Bernstein, 1994), indicates that the choice of reliability estimator does not affect the conclusion (Revelle & Zinbarg, 2009).

### 3.3. Discriminant Validity

Discriminant validity was examined using the Fornell–Larcker criterion (Fornell & Larcker, 1981) and the heterotrait–monotrait ratio (HTMT; Henseler et al., 2015), a procedure recommended for combined use because the first criterion loses sensitivity when loadings are homogeneous, as is the case with these data (Voorhees et al., 2016); both criteria were met without exception (Table 7). The square root of the average variance extracted for each construct, ranging from 0.749 to 0.769, exceeded any correlation with the other constructs, the maximum magnitude of which was 0.625 between AI literacy and self-regulated learning. The heterotrait–monotrait ratio reached a maximum of 0.608 for that same pair, well below the strict threshold of 0.85 proposed for conceptually closely related constructs (Henseler et al., 2015). It should be noted that the ratio between dimensions of the same construct does exceed this threshold, with values as high as 0.913; these are facets of the same higher-order factor, and the discriminant criterion applies between constructs, not between facets.

### 3.4. Measurement Invariance

The configural-metric-scalar sequence was tested according to the protocol established by Vandenberg and Lance (2000), and decisions were based on the CFI differential using the criterion ΔCFI ≤ 0.010 (Cheung & Rensvold, 2002), which is more appropriate than the chi-square test when the sample is large (Meade et al., 2008). By gender, the measurement structure was found to be fully invariant (Table 8). None of the successive restrictions significantly worsened the fit: the comparison between the metric and configural models yielded Δχ^2^ (43) = 39.30, *p* = 0.632, and the comparison between the scalar model and the metric model yielded Δχ^2^ (137) = 110.83, *p* = 0.951, with CFI differentials of equal to or less than 0.001. The comparisons between men and women regarding the metrics of the constructs are therefore interpretable.

By type of university, metric invariance was comfortably supported, Δχ^2^ (43) = 50.96, *p* = 0.189. The scalar restriction resulted in a statistically significant deterioration, Δχ^2^ (137) = 172.95, *p* = 0.020, but of negligible practical significance: the CFI did not change, and the RMSEA remained at 0.020. With 820 cases and 2236 degrees of freedom, the chi-square test detects discrepancies without substantive consequences a sensitivity to sample size noted in early work on incremental indices (Bentler & Bonett, 1980) and confirmed in simulation studies on invariance (Cheung & Rensvold, 2002; Meade et al., 2008). Therefore, scalar invariance is assumed in a practical sense, and this qualification should be noted when interpreting comparisons of means between the two types of institutions.

### 3.5. Common-Method Bias

Common-method bias was examined in three ways, following the diagnostic and control recommendations of Podsakoff et al. (2003, 2012). The unifactorial model, a confirmatory version of Harman’s (1967) test, showed poor fit (CFI = 0.549, RMSEA = 0.146), which argues against a single general factor accounting for the covariance; it is nevertheless reported as preliminary evidence, given its low sensitivity for detecting bias when it is moderate (Fuller et al., 2016). The model with an unmeasured latent method factor, a procedure proposed by Liang et al. (2007) in line with the uncorrelated markers approach (Lindell & Whitney, 2001), loading on the 47 categorical items, did not achieve convergence within the iteration limit, a predictable result given the computational demands of estimating 47 additional loadings on ordinal indicators using WLSMV. Its equivalent model based on the 14 dimension scores did converge and provides supporting evidence: the mean variance attributable to the method factor was 3.6%, compared to the 70.5% explained by the substantive factors, well below the 25% threshold for concern established in the literature on method variance (Podsakoff et al., 2003, 2012). Incorporating the method factor also did not improve the model’s fit according to information, with AIC = 22,566.2 compared to 22,554.0 for the model without it. Two qualifications bound what these diagnostics establish. The item-level method-factor model did not converge, so the diagnostic that did converge was estimated on the fourteen dimension scores rather than on the 47 items, and an aggregated diagnostic is less sensitive to method effects that operate at item level. And all four constructs were reported by the same respondents, in a single session, using a common response format, which is the configuration in which method variance is most likely to arise. The appropriate conclusion is therefore the narrower one: the available statistical diagnostics did not indicate a dominant common-method factor. That is not the same as establishing that common-method variance is negligible, and the results should be read with that residual uncertainty in view.

### 3.6. Structural Model and Testing of Mediation

The mediation model without interaction terms reproduced the expected pattern (Table 9 and Table 10). AI literacy substantially predicted self-regulated learning, *b* = 0.510, *SE* = 0.029, β = 0.626, *p* < 0.001, and both literacy, β = 0.243, and self-regulated learning, β = 0.445, predicted the responsible use of generative AI. Technostress showed a negative main effect on responsible use, β = −0.195, *p* < 0.001, but no effect on self-regulated learning, β = 0.031, *p* = 0.370. The model explained 38.5% of the variance in self-regulated learning, *R*^2^ = 0.385, and 46.6% of the variance in responsible use, *R*^2^ = 0.466, *proportions that* fall within the moderate-to-substantial range of the scales available for structural equation models in the behavioral sciences (Chin, 1998; Cohen, 1988).

The indirect effect of AI literacy on responsible use via self-regulated learning was significant, *b* = 0.217, *SE* = 0.023, β = 0.278, 95% CI [0.175, 0.265] for the unstandardized coefficient and 95% CI [0.226, 0.337] for the standardized coefficient; both intervals, corrected for bias and obtained from 10,000 resamples, exclude zero, a procedure preferable to the Sobel test because it does not assume normality of the coefficient product distribution (Hayes, 2022; Preacher & Hayes, 2008). The direct effect remained significant, *b* = 0.189, β = 0.243, 95% CI [0.123, 0.258]: the indirect path accounts for 53.4% of the total effect, *b* = 0.406, β = 0.522. The pattern therefore corresponds to partial mediation, a label that here describes the distribution of the effect and does not constitute a substantive conclusion, given its dependence on sample size (Rucker et al., 2011); the inference relies on the bootstrap interval of the indirect effect rather than on the sequence of causal steps in the classical approach (Baron & Kenny, 1986), which has been superseded by resampling procedures (MacKinnon, 2008; Preacher & Hayes, 2008). The hypothesis regarding the mediating role of self-regulated learning is supported, and the magnitude of the standardized indirect effect, β = 0.278, corresponds to a medium effect size according to conventional standards (Cohen, 1988, 1992) and is of considerable practical relevance in applied research (Funder & Ozer, 2019).

The coefficients behind these correlations are reported separately. Table 10 breaks down each path of the mediation model with its unstandardized and standardized estimate, its standard error, and its bootstrap confidence interval.

### 3.7. Moderated Mediation

The inclusion of latent interaction terms, specified on centered variables to preserve the interpretability of conditional effects (L. S. Aiken & West, 1991; Cohen et al., 2003), significantly improved the fit. The scaled log-likelihood difference test compared to the model without interactions yielded TRd = 41.11, *gl* = 3, *p* < 0.001, and both information criteria favored the model with interactions: AIC = 22,521.5 versus 22,554.0, and BIC = 22,761.7 versus 22,780.1. Technostress therefore moderates some of the model’s pathways.

**Figure 2:**
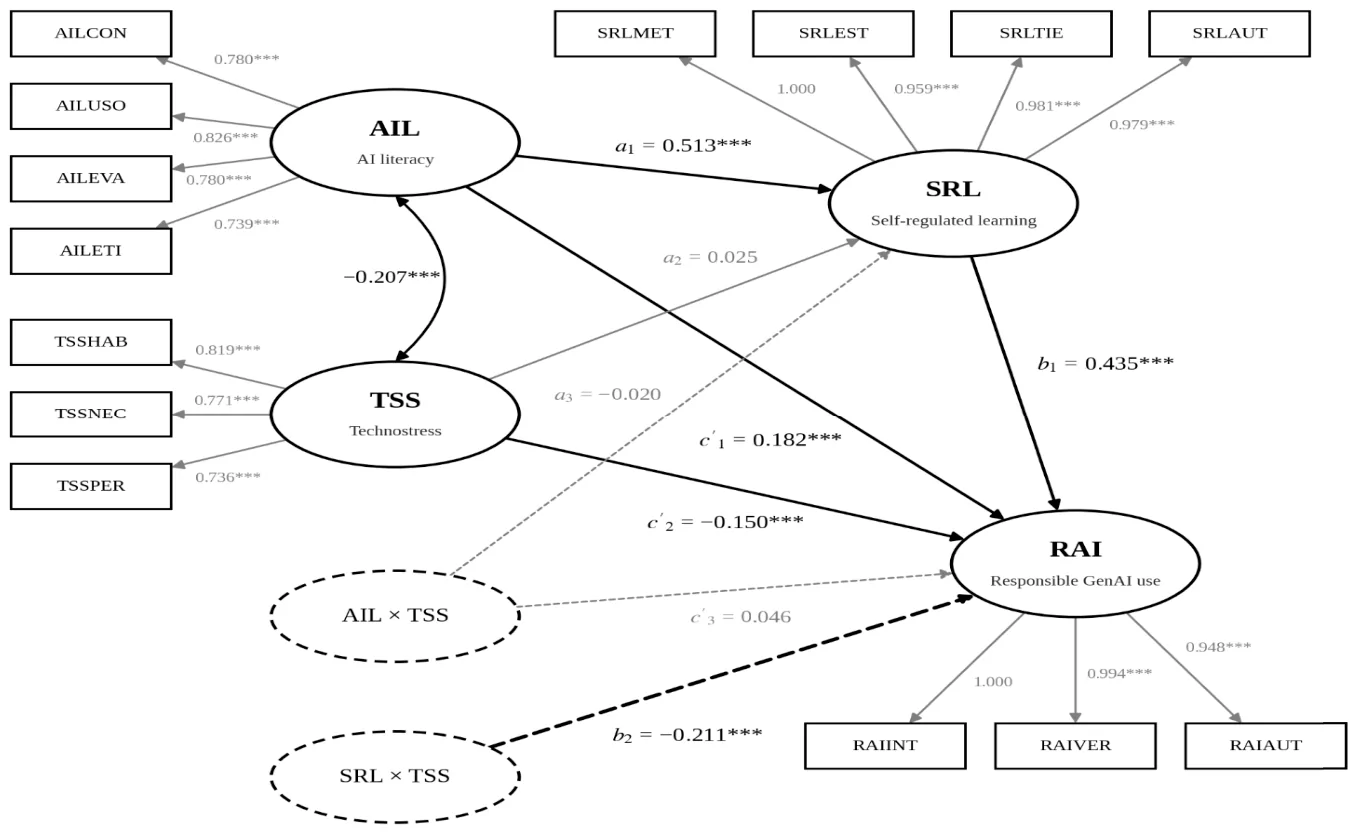
Two-stage moderated mediation with moderation of the direct effect. *Note. N* = 820. AIL = artificial intelligence literacy; SRL = self-regulated learning; TSS = technostress; RAI = responsible use of generative AI. The ellipses represent latent variables, and the rectangles represent the observed indicators, which correspond to the 14 dimension scores described in Table 6. The double-headed curved arrow indicates the covariance between the two exogenous variables.

**Figure 3:**
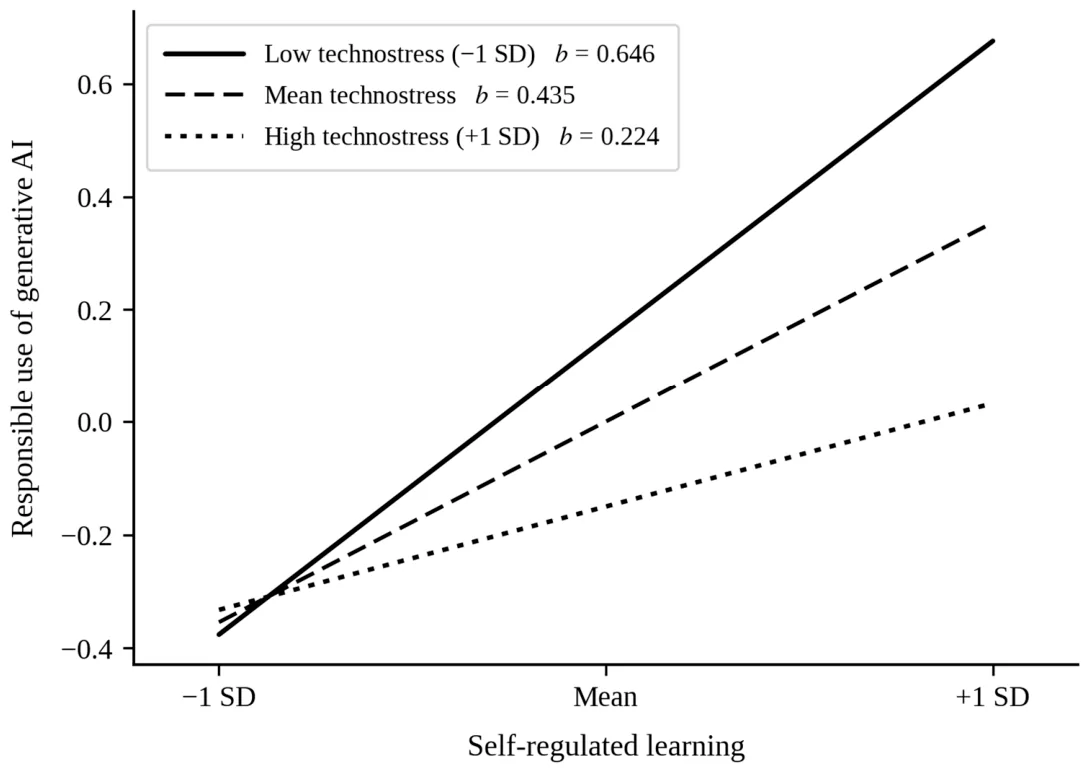
Simple Slopes of Self-Regulated Learning Regarding the Responsible Use of Generative AI According to the Level of Technostress. *Note. N* = 820. The slopes were estimated at −1 standard deviation, the mean, and +1 standard deviation of latent technostress. Responsible use of generative AI is expressed as deviations from its latent mean.

Of the three pathways subjected to moderation, only one proved significant (Table 11 and Figure 2). The interaction on the first stage was not significant, *a*_3_ = −0.020, *SE* = 0.026, *p* = 0.440, and neither was the interaction on the direct pathway, *c′*_3_ = 0.046, *p* = 0.182. The interaction on the second stage, however, was significant and negative, *b*_2_ = −0.211, *SE* = 0.040, *p* < 0.001. Technostress was not associated with the strength of the link between AI literacy and self-regulation; it was associated with a weaker link between self-regulation and self-reported responsible use. The effect of self-regulated learning on responsible use ranges from *b* = 0.646 when technostress is one standard deviation below the mean to *b* = 0.224 when it is one standard deviation above, a slope nearly two-thirds smaller at high than at low technostress (Figure 3). Simple slopes were estimated and plotted at the mean and at ±1 standard deviation of the moderator, in accordance with the conventional procedure for testing interactions (L. S. Aiken & West, 1991; Dawson, 2014).

The values next to the measurement arrows are fully standardized loadings; those next to the structural paths are unstandardized coefficients. AIL and TSS were scaled by fixing their latent variance at 1. The thick dashed line indicates significant interaction, and the thin dotted line indicates the two nonsignificant interactions. *** *p* < 0.001; the coefficients shown without asterisks were not statistically significant.

The size of this attenuation is easier to read graphically. Figure 3 plots the slope of self-regulated learning on responsible use at three levels of technostress, which makes clear the flattening described above.

The parameters underlying both figures are reported below. Table 11 brings together the structural coefficients, the three interaction terms, and the conditional effects evaluated at the levels of the moderator.

**Figure 4:**
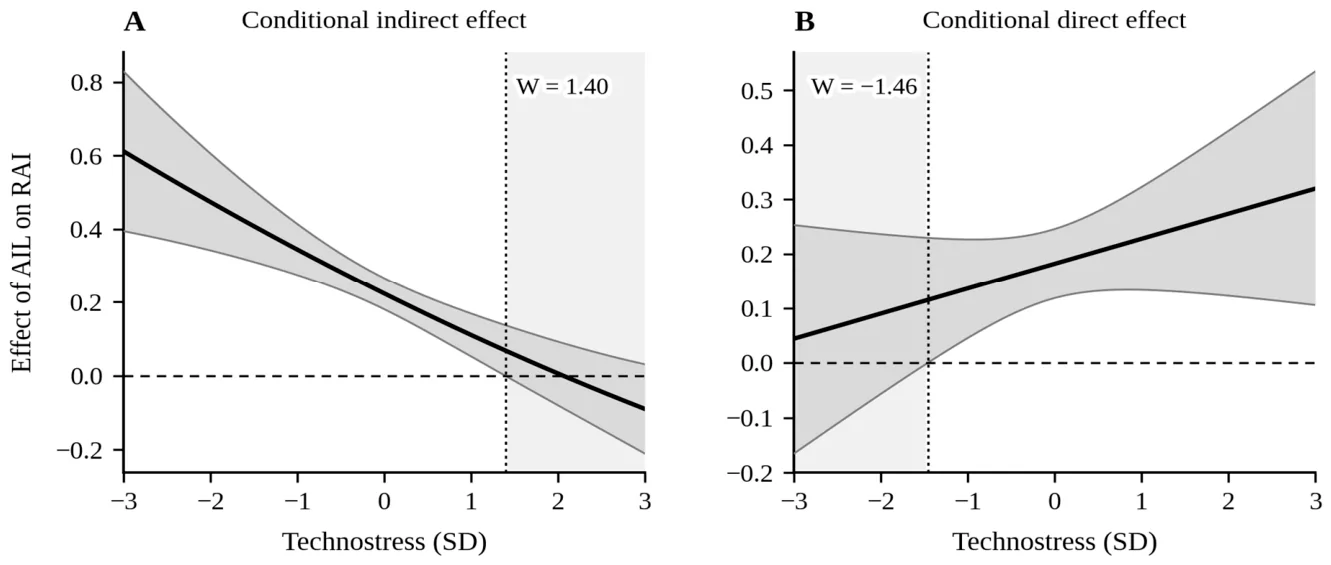
Conditional effects of AI literacy on responsible use along the technostress continuum. *Note. N* = 820. (**A**) shows the indirect effect via self-regulated learning, and (**B**) shows the direct effect. The gray band is the 95% confidence interval obtained using the delta method; the shaded area indicates the range of the moderator within which the effect is not statistically significant, bounded by the estimated Johnson–Neyman region-of-significance boundary. *W* = latent technostress in standard deviations.

The conditional indirect effects reflect this pattern. When technostress is one standard deviation below the mean, the indirect effect of AI literacy on responsible use is 0.344, *SE* = 0.036; at the mean, it is 0.223; and when it is one standard deviation above the mean, it is 0.111. The difference between the extreme levels is significant, −0.233, *SE* = 0.048, *p* < 0.001, supporting moderated mediation. The Johnson–Neyman region of significance analysis, which avoids the arbitrariness of testing the moderator only at discrete values (Hayes, 2022; Spiller et al., 2013), places the estimated region-of-significance boundary at *W* = +1.40 standard deviations (Figure 4): above that estimated boundary, the indirect effect is not statistically distinguishable from zero in this sample. The boundary is a sample estimate with its own sampling variability, not a fixed threshold or a tipping point in the process, and the cross-sectional design gives it no temporal meaning. A conditional indirect effect that is not statistically distinguishable from zero is also not an estimate of zero: the absence of significance beyond this boundary reflects the precision available in this sample at those levels of the moderator and does not establish that the association ceases to exist there.

The direct effect followed the opposite pattern, although its interaction term was not significant: it increased from 0.137 to 0.228 between the extreme levels of the moderator, with a difference that did not reach significance, 0.092, *p* = 0.182. The interaction term was not significant, so this pattern is not evidence that under high technostress AI literacy operates through mechanisms other than self-regulation. That possibility is raised only as a direction for future research and would require a design built to test it.

### 3.8. Robustness Analysis

The pattern is held in both specifications with covariates (Table S3 in the Supplementary Material). With controls for the mediator and the outcome, the second-stage interaction barely changed, b2 = −0.212, *p* < 0.001, and the first stage and direct-path interactions continued to fail to reach significance. With full control, which also residualizes the exogenous variables, the coefficients were attenuated, as expected, but retained their sign and significance: the second-order interaction remained at −0.205, *p* < 0.001, and the difference between extreme indirect effects at −0.185, *p* < 0.001. In this more heavily adjusted specification, the indirect effect under high technostress was not statistically significant, 0.062, *p* = 0.065, which is consistent with the substantive conclusion. In summary, the results do not appear to depend on the sociodemographic composition of the sample. The stability of the sign, significance, and order of magnitude of the coefficients across three nested specifications constitutes the evidence of robustness expected from an interpretable structural model (Kline, 2023), and the attenuation observed under full control should be interpreted in terms of effect size rather than a mere significant–nonsignificant dichotomy (Funder & Ozer, 2019; Lakens, 2013).

A further check addresses the composition of the analytic sample by excluding the 81 respondents who reported no use of generative AI for academic work during the survey period (Table 2), all of whom met the eligibility criterion of prior experience but whose inclusion a reader might question. Re-estimating the model on the remaining 739 cases leaves every conclusion intact: AI literacy predicted self-regulated learning, β = 0.616 against 0.626 in the full sample, and responsible use, β = 0.246 against 0.243; self-regulated learning predicted responsible use, β = 0.426 against 0.445; the indirect effect remained significant, β = 0.262 against 0.278, accounting for 51.6% of the total effect against 53.4%. The moderation pattern is likewise unchanged: the second-stage interaction remained negative and significant, *b*_2_ = −0.178 against −0.182 in the full sample estimated on the same metric, *p* < 0.001, while the first-stage and direct-path interactions remained non-significant, *a*_3_ = −0.028, *p* = 0.358, and *c′*_3_ = 0.023, *p* = 0.495. The difference between the conditional indirect effects at the extreme levels of the moderator remained significant, −0.222, 95% CI [−0.312, −0.131], against −0.229, 95% CI [−0.313, −0.142]. The eligibility question raised by the frequency distribution therefore does not account for the reported findings.

Three checks respond to concerns about what the model’s constructs capture. The first concerns institutional policy. Only 17.1% of respondents reported that their university had an AI policy, while 37.3% reported none and 45.6% did not know, which raises the question of whether the normative integrity items were answerable for the majority. Three indications suggest they were. The dimension was internally consistent in all three groups (α = 0.821 where no policy exists, 0.870 where the student is unaware, 0.860 where a policy exists), so respondents were not answering at random. Its mean rose monotonically with policy awareness (M = 3.371, 3.526 and 3.837 respectively), which is the ordering expected if the items track a real difference in normative context rather than measurement noise. And the model reproduced in each group: the indirect association was 0.222 where no policy exists, 0.305 where the student is unaware and 0.285 where a policy exists, with AI literacy predicting self-regulated learning at β = 0.617, 0.618 and 0.587. Removing normative integrity from the outcome altogether, so that responsible use is defined by verification and authorship alone, leaves the estimates essentially unchanged (β = 0.626 for the first stage, 0.453 for the second, indirect 0.284 against 0.278). The conclusions do not rest on the normative integrity items.

The second check addresses the ethical content shared by the predictor and the outcome. Removing the ethics facet from AI literacy, so that the construct is defined by awareness, use and evaluation only, changes nothing of substance: AI literacy still predicts self-regulated learning (β = 0.621 against 0.626) and self-reported responsible use (β = 0.236 against 0.243), self-regulated learning still predicts the outcome (β = 0.450 against 0.445), and the indirect association is unchanged (β = 0.279 against 0.278). The facet-level correlations point the same way. The ethics facet correlates with the three responsible-use facets at r = 0.415 on average, which is no higher than the corresponding figures for awareness (0.408), use (0.420) or evaluation (0.410). Were the association driven by overlapping item content, the ethics facet should stand out; it does not.

The third check concerns the independence of observations. Institutional identifiers were not retained (Section 2.2), so clustering cannot be modelled directly, and what follows is partial evidence rather than a substitute for cluster-robust estimation. Across the stratification variables that were retained, between-group variance is negligible: the intraclass correlation across the five fields of study is below 0.001 for all four constructs, and across the four university-type by region cells it reaches 0.020 for technostress and 0.004 for responsible use, with AI literacy and self-regulated learning at zero. Design effects of this magnitude would leave standard errors substantively unaffected. This is reassuring at the level of the strata that were recorded and silent about clustering within individual institutions or classrooms, which Section 4.7 records as a limitation.

A final check bears on how the two non-significant interactions should be described. Failure to reject a null hypothesis is not evidence of equivalence, so the interactions for H5 and H7 were submitted to two one-sided tests against equivalence bounds of ±0.10 in the latent metric, a bound corresponding to the smallest interaction the design was powered to detect. The first-stage interaction is statistically equivalent to zero, *a*_3_ = −0.020, 90% CI [−0.063, 0.023], *p* = 0.001, and the same holds in the composite metric, *p* = 0.004. The direct-path interaction is not: *c′*_3_ = 0.046, 90% CI [−0.010, 0.102], *p* = 0.056, with the upper bound marginally outside the equivalence region. H5 may therefore be described as an effect statistically equivalent to zero within the bound tested, whereas H7 supports only the weaker statement that no significant moderation was detected. The manuscript adopts that distinction throughout.

### 3.9. Summary of Hypothesis Testing

Six of the eight hypotheses were supported (Table 12). The two that were not supported share a common feature: both attributed a moderating role to technostress on pathways originating from AI literacy. No significant moderation was detected on the path from AI literacy to self-regulation or on the direct path; the moderation that was detected falls at the final stage, where self-regulation is associated with self-reported responsible behaviour. As Section 4.3 sets out, a non-significant interaction is not evidence that technostress does not moderate those paths.

## 4. Discussion

### 4.1. Summary and Conclusions of the Model

Prior to this study, Peruvian evidence on generative artificial intelligence in higher education relied on bivariate associations and single-stage models linking technological competence to cognitive outcomes, without specifying the process through which competence may be associated with behavior or the conditions under which that association is weaker. This study tested a two-stage moderated mediation model with latent variables on 820 students. The result is mixed in an informative sense: the proposed pattern of association held, while significant moderation was detected on only one of the three paths for which the theory predicted it. The two unsupported hypotheses (Section 3.9) share the feature that underpins this discussion, as both attributed to technostress a conditioning role in pathways stemming from AI literacy.

### 4.2. Self-Regulation as a Mediating Pathway

Artificial intelligence literacy predicted self-regulated learning with the highest coefficient in the model (β = 0.626), a result that aligns with the direction established by the three-level meta-analysis of 95 effect sizes by J. Wu et al. (2026) and with Peruvian evidence that self-efficacy in AI learning is the strongest predictor of technology adoption (Acosta-Enriquez et al., 2025a). This convergence is noteworthy for its nuance: those studies measured interventions or efficacy beliefs, whereas here we measured a self-reported multidimensional competence, and the relationship held. Understanding what a generative system can and cannot do appears to facilitate one’s own study planning, not replace it.

Self-regulated learning, in turn, predicted self-reported responsible use (β = 0.445), and literacy retained its own direct association (β = 0.243). The indirect effect was significant with a bias-corrected bootstrap interval and accounted for 53.4% of the total effect, while the direct effect remained significant. This distribution of the effect deserves full discussion rather than a footnote. Bandura (1991) does not argue that all behavior involves self-regulatory deliberation, and the responsible use of generative AI includes components of normative compliance that a student may carry out based on knowledge of the rule, without any strategic process whatsoever. The fact that approximately half of the effect is mediated by self-regulation and the other half is not consistent with a dependent construct that combines low-cost acts such as adhering to a known norm with deliberate and costly acts, such as verifying a generative output or declaring one’s contribution.

This result qualifies two previous Peruvian findings without contradicting them. Vereau Amaya et al. (2025) concluded, based on a sample of 200 first-year students, that unmediated use of AI could displace self-regulatory processes, and García Castro et al. (2026) described a shift toward cognitive delegation based on 265 interviews. Both observed the reverse of the pattern estimated here: where self-regulation does not intervene, use is associated with dependence. The evidence thus converges on the idea that self-regulation is the critical link, and this study provides an estimate of the missing piece. The pattern is also consistent with the metacognitive laziness described by Fan et al. (2025), who found that working with ChatGPT improves the output without improving the process: if the process is the pathway along which competence is associated with responsible behaviour, a use that does not activate it would not be expected to show that association, however much it raises the quality of the work delivered.

### 4.3. Where Technostress Comes into Play

The distinctive finding of the study is asymmetrical. No significant moderation by technostress was detected on the association between AI literacy and self-regulation (*a*_3_ = −0.020, *p* = 0.440) or on the direct path (*c′*_3_ = 0.046, *p* = 0.182), whereas technostress moderated, substantially and negatively, the association between self-regulation and self-reported responsible behavior (*b*_2_ = −0.211, *p* < 0.001). The slope of self-regulated learning regarding responsible use was nearly two-thirds smaller at the high than at the low level of the moderator. This contrast between stages, formalized in the moderated path analysis by Edwards and Lambert (2007), is what gives meaning to a body of evidence that until now had seemed inconsistent.

The null result from the first stage replicates, using a different model and latent estimation, what Marquez-Yauri et al. (2026) found among 340 Peruvian college students: no direct effect of technostress on the cognitive outcome and no support for its moderating role in the relationship between self-efficacy in AI and critical thinking. Those authors interpreted this null result as evidence that technostress is a distal correlate. The data from this study suggest a more precise interpretation: technostress may be localized rather than distal. Stage-specific null results of this type are common in the technostress literature and are not anomalous. H. Wu et al. (2026) found that the interaction between AI anxiety and literacy predicted self-efficacy but not dependence via the direct path; Di Stefano et al. (2025) observed significant moderation on the X→M path that did not extend to the moderated mediation index; and Wirth et al. (2024) found that employer support moderated only one of the five technostress indicators assessed. Partial moderation appears to be the rule in this domain, not the exception.

It is advisable not to present the null result as definitive, because two recent studies did find moderating effects that were not observed here. Türk et al. (2025) found among 356 Turkish college students that anxiety about learning AI attenuates the direct path between attitude toward AI and acceptance of generative systems, and Duong et al. (2025) documented among 2602 users that technology anxiety moderates both the direct and indirect paths in a technostress model. Three differences could account for this divergence without invoking chance. The first is construct-related: both studies measured technology anxiety, an anticipatory and reactive state associated with the act of using the tool, while this study measured technostress as cumulative person–environment mismatch (Edwards et al., 1998), a more stable condition. The second is related to the outcome: those studies examined technology acceptance and life satisfaction, not norm-referenced academic behavior, and there is no reason to expect a moderator to act the same way on outcomes of such different natures. The third is related to aggregation: Yang and Tang (2026) demonstrated in a sample of 649 students that techno stressors associated with generative AI produce effects of opposite signs depending on the stressor, and the distinction between challenge and obstacle stressors precisely predicts this divergence (Cavanaugh et al., 2000; LePine et al., 2005), with recent confirmation in online learning environments (Lallmahomed, 2025). An aggregated measure such as the one used here could in principle cancel out mutually offsetting effects in the first stage and reveal only the net effect in the stage where the hindrance component predominates. This account was not tested in the present study, which measured technostress as a single aggregate and therefore cannot separate challenge from hindrance components; it is a candidate explanation of the first-stage null result and a specific hypothesis for future research, not a conclusion supported by these data. Testing it requires an instrument that scores the two stressor types separately, which is proposed in Section 4.7.

The theoretical interpretation of the second-stage effect follows the logic of resource conservation. A constraint on the reading of this result should be registered before the interpretation is developed. Technostress was measured as a general person–environment technological mismatch, not as stress attributable to generative systems, so what the data support is an interaction between general technostress and self-reported responsible AI-use practices. They do not identify generative AI as the source of the stress, and no conclusion about GenAI-specific stress follows from them. The interpretation below should be read as an account of how general technological strain relates to a specific set of academic practices. Verifying a generative output against independent sources and declaring its contribution in a deliverable are deliberate, time-consuming acts carried out under time pressure, precisely the type of investment that is cut back first when a person perceives a threat to their reserves (Hobfoll, 1989) and when sustained demand displaces the motivation and attention that sustain regulatory effort (Inzlicht & Schmeichel, 2012). Setting goals and planning the study, on the other hand, are operations that precede the task and compete less with immediate urgency. The asymmetry observed is the pattern that would be expected if technostress raised the cost of performing the behaviour without affecting the capacity to form it. The cross-sectional design does not permit this account to be tested, and it is offered as an interpretation to be examined in future work rather than as a demonstrated process.

The direct effect followed the opposite pattern, although the difference between the extreme levels of the moderator did not reach significance (Section 3.7). One reading of this pattern is a retreat toward normative compliance when deliberation becomes more costly, but the interaction term was not significant and the study contains no test of such a retreat. The pattern is reported for completeness and the interpretation is offered as a conjecture for future work, not as a finding. The distinction between deliberative and heuristic processing offers a plausible framework for this interpretation (Evans & Stanovich, 2013), and Deng et al. (2026) documented a compensatory use of AI precisely under obstacle stressors. Testing this possibility would require a design specifically tailored for that purpose, and here it can only be noted as a derived hypothesis.

### 4.4. The Conditional Nature of the Indirect Association

The formal test of moderated mediation, which assesses whether the indirect effect varies with the level of the moderator rather than inferring it from the significance of the interaction terms separately (Preacher et al., 2007), proved significant. The indirect effect was roughly two-thirds smaller at high than at low levels of the moderator (Section 3.7), and the estimated region-of-significance boundary was located at 1.40 standard deviations above the mean. If the latent moderator is normally distributed, about 8% of students would score above that estimated boundary; that is, approximately one in twelve. For that subgroup, the indirect association between AI literacy and responsible use through self-regulation was not statistically distinguishable from zero in this sample, which does not establish that the association is absent.

The robustness specification is consistent with this reading. Under full control, which also accounts for exogenous variables, the indirect effect at high technostress was not statistically significant (0.062, *p* = 0.065), while the second-stage interaction remained significant (−0.205, *p* < 0.001). That the region of non-significance begins at a lower level of the moderator once sociodemographic composition is controlled suggests that the estimated boundary is unlikely to be an artefact of the sample profile. It remains an estimated boundary rather than a threshold: it is conditional on this sample, on the aggregated technostress measure, and on the cross-sectional design, and it should not be read as a point at which a process switches off. This pattern contrasts with that of Di Stefano et al. (2025), where the moderation of a path failed to produce a significant moderated mediation index, and aligns with that of Kong et al. (2026), who specified and found moderation exclusively in the second stage of a mediated chain regarding problematic use of generative AI.

### 4.5. Theoretical Implications

The first implication refines social cognitive theory in a specific regard. Bandura (1986) argues that personal factors influence behavior through agency processes and that the environment restricts this influence but does not specify at what point in the cycle the restriction occurs. The results are consistent with adverse environmental conditions being associated less with the formation of self-regulatory agency than with its behavioral execution. This distinction between forming and exercising is not a semantic nuance: if confirmed by longitudinal or experimental designs, it would imply that interventions seeking to promote responsible behavior in the face of technological stress should target the conditions of execution, not the teaching of regulatory strategies, which stress does not appear to block.

The second implication concerns the literature on technostress. Treating technostress as an undifferentiated main effect has produced contradictory results that this study helps to clarify. Here, technostress showed a negative main effect on responsible use (β = −0.195) and no significant association with self-regulated learning (β = 0.031, *p* = 0.370), a pattern that is consistent with its interpretation as an execution condition. This leads to a recommendation for the design of future models: specify the stage prior to estimation, using the moderated path framework of Edwards and Lambert (2007), and do not introduce the moderator globally.

The third implication concerns artificial intelligence literacy. Recent reviews agree on incorporating ethical responsibility as a component of the construct (Park, 2025; Zainal et al., 2026), and the data support that this competency is associated with responsible behavior. However, the association between literacy and self-reported behavior was conditional in these data, and this conditionality is not reflected in the available frameworks, which treat literacy as a capacity whose effect is assumed to be constant. The indirect association of literacy with responsible use was considerably stronger at low than at high levels of technostress, suggesting that frameworks should incorporate conditions of application alongside competencies.

### 4.6. Practical Implications

The most direct implication is aimed at universities and follows from the second stage finding. If technological pressure makes verification and authorship declaration more costly, institutional policy should reduce the cost of these behaviors rather than multiply the rules that require them. In operational terms, this means incorporating the AI usage disclosure field directly into the submission platform itself, rather than leaving it as an attachment that students must remember to include and draft, and providing brief checklists integrated into the assignment instructions. Evidence on barriers to disclosure supports this approach: in a sample of students, only 9% always disclosed their use of generative AI, and the predominant reasons were fear of peer judgment and faculty sanctions, along with the belief that disclosure is equivalent to admitting wrongdoing (Adnin et al., 2025). A standardized, low-effort disclosure procedure could address all three barriers at once.

The second implication concerns the design of training. Artificial intelligence literacy programs are necessary, given that competence predicted both self-regulation and responsible behavior, but they may not suffice on their own for the subgroup with high technostress, in which the indirect association through self-regulation was not statistically distinguishable from zero. For this segment, the available evidence points to task-integrated metacognitive scaffolding, which improved strategy and self-assessment in generative environments (Xu et al., 2025) and increased self-efficacy by reducing learning anxiety (Jiang & Xu, 2026). Skills training and process scaffolding serve distinct functions and are not interchangeable.

The third implication concerns institutional support as a buffer. Instructor support and institutional support significantly mitigate the effect of technostress on the quality of online learning (Saleem et al., 2024), and the framework of technostress inhibitors suggests that stress is partly a design issue and not solely a matter of individual disposition (Tarafdar et al., 2019). The contextual data from this sample points to an urgent recommendation: 37.3% of students reported that their university has no policy on the use of AI, and another 45.6% are unaware of whether such a policy exists, which aligns with the policy vacuum documented in Peruvian universities (Montoya-Cantoral et al., 2023). In this context, formulating and communicating the policy could serve as a measure to reduce technostress rather than as an act of control.

### 4.7. Limitations and Future Directions

The cross-sectional design precludes making causal claims; in particular, estimating mediation based on data from a single time point may skew the coefficients compared to those that would be obtained with longitudinal data, in directions and magnitudes that are not always predictable (Maxwell & Cole, 2007; Maxwell et al., 2011). The reported relationships should be interpreted as structural associations consistent with the model, not as established temporal sequences. This qualification applies to the mediated and the conditional estimates alike: a cross-sectional indirect effect is not evidence that self-regulation intervenes between literacy and behaviour over time, and a cross-sectional conditional indirect effect is not evidence that technostress alters that process as it unfolds. Both are associations whose magnitude depends on the level of a third variable measured simultaneously. A relevant future line of research would be a repeated-measures design that estimates mediation with a time lag between literacy, self-regulation, and behavior. The design is also compatible with directions of association other than the one modelled. Students with stronger self-regulatory habits may become more AI literate rather than AI literacy, giving rise to self-regulation, and students who already verify and disclose may develop both competences as a consequence of that practice. Nothing in these data adjudicates between the modelled ordering and its alternatives. The model is offered as one specification consistent with social cognitive theory, and the ordering it assumes remains an assumption of the specification rather than a finding of the study.

All constructs are self-reported, which introduces a risk of common-method bias and, in the case of responsible use, social desirability bias. The available statistical diagnostics did not indicate a dominant common-method factor, with 3.6% of the variance attributable to the method factor compared to 70.5% explained by substantive factors in the dimension-score model, whose item-level counterpart did not converge; however, direct self-report tends to underestimate academically questionable behavior compared to protected response techniques (Scheers & Dayton, 1987; Tourangeau & Yan, 2007), and the discrepancy between acknowledged use and self-reported use has already been documented among Peruvian college students (Oré Gálvez et al., 2026). The future agenda involves behavioral measures: effective inclusion of usage disclosures in submitted papers, verification traces in the writing process, or randomized response designs.

Technostress was measured as a general person–environment mismatch rather than as stress specific to generative systems. Given that techno stressors associated with generative AI produce effects of varying nature depending on their specific characteristics (Lallmahomed, 2025; Yang & Tang, 2026), a disaggregated measure by stressor type could reveal, in the initial stage, effects that the aggregate measure obscures. Replication using specific instruments for technostress related to generative AI and specifying challenge and obstacle stressors is a natural extension of this study. The same caution applies to the conclusions drawn from the moderation: they concern general technological strain and not stress attributable to generative systems, and they should not be read as identifying generative AI as the source of the strain.

The sampling was non-probabilistic and limited to Peruvian undergraduate students; therefore, generalization to other populations requires caution, even though the measurement structure proved to be invariant across gender and, in practical terms, across university types. Comparative studies between countries with different levels of institutional regulatory maturity would help determine whether the estimated region-of-significance boundary shifts when regulations exist and are known. Finally, the scale for the responsible use of generative AI was developed for this study, and despite its satisfactory psychometric performance, it requires external validation with independent samples before its scores can be compared across contexts. The evidence reported here is internal to a single sample and to the panel that reviewed the items, and it does not substitute for independent validation by research teams that were not involved in developing the instrument. The scale should therefore be treated as a provisional operationalisation offered for replication rather than as a validated measure.

Two features of the design constrain the reported standard errors and the reported outcome in ways that could not be addressed analytically. Recruitment through classrooms and institutional networks makes clustering within universities, courses and referral chains likely, and the anonymisation protocol did not retain the identifiers needed to model it, so the standard errors reported here assume an independence that the sampling design does not guarantee. Mode of administration was likewise not retained, so it could be neither adjusted for nor tested for measurement invariance, even though supervised and unsupervised administration may plausibly elicit different levels of reporting on items about academic integrity. Both are limitations of the data as archived rather than points open to further analysis, and both argue for recording cluster and mode identifiers in replications. Table 13 sets out how the findings of this study converge with or diverge from the previous literature for each hypothesis.

## 5. Conclusions

This study addressed a gap that the Peruvian literature on artificial intelligence in higher education had left open on three fronts. The process through which proficiency in artificial intelligence may be associated with responsible use had not been modeled; the role of technostress had been examined as a main effect or as a moderator of cognitive pathways without empirical support; and responsible use lacked an operationalization that distinguished its components. The moderated mediation model estimated across 820 students addresses all three: identifies self-regulated learning as a pathway that statistically accounts for approximately half of the association between literacy and self-reported behavior, locates the stage of the model at which moderation by technostress was detected, and provides a three-dimensional measure of responsible use with evidence of reliability, validity, and invariance.

The central finding can be stated without resorting to statistics. Technostress was unrelated to self-regulation among students with greater proficiency in artificial intelligence, and no significant moderation was detected on the direct path to their reported compliance with rules they already know. The association that is weaker at higher technostress is the one at the final stage, where self-regulation would be expressed as verifying what the tool produces and declaring one’s own participation in the work. Where technostress is high, these two behaviours—the most demanding in the repertoire of responsible conduct—are the ones reported least often. Because the data are cross-sectional, this ordering describes a pattern of association and not a sequence established over time. Above the estimated region-of-significance boundary of +1.40 standard deviations of technostress, the indirect association was not statistically distinguishable from zero in this sample, which suggests that for students in that range skills training alone may not be sufficient. The value is an estimated region-of-significance boundary carrying its own uncertainty, not a threshold at which the process stops.

Hence the practical takeaway. Literacy appears to matter, given its association with self-reported responsible use, but the strength of its indirect association through self-regulation varied with students’ level of technostress. In a university system where most students report that there are no institutional guidelines on the use of AI or that they are unaware of them, a promising intervention would involve making compliance more accessible rather than adding requirements, by integrating authorship declarations and content verification into the submission workflow itself, and by supplementing skills training with process scaffolding for the subgroup under the greatest stress.

The conclusions must be interpreted within the limits of the study design. The data are cross-sectional and self-reported; the sample is non-probabilistic; and technostress was measured in aggregate form. Consequently, the temporal order of the process remains to be established; reported behavior may differ from observed behavior; and the absence of moderation in the first stage may reflect the cancellation of effects between types of stressors rather than their absence. Each of these limitations opens a specific line of research: longitudinal designs with time-lagged measurements, behavioral indicators involving self-reporting and verification, generative AI-specific technostress instruments with disaggregated stressors, and comparative replications across contexts with varying levels of regulatory maturity.

There is a broader lesson that transcends the Peruvian case. Public discourse on artificial intelligence in universities is often framed as a matter of knowing versus not knowing, and the typical institutional response consists of teaching and regulating. The results suggest that the breaking point lies neither in knowledge nor in the willingness to comply, but rather in the cost of doing so when technology has already become a source of tension. Designing academic environments where responsible behavior is low-cost may yield better results than insisting on explaining why it is advisable.

## Figures and Tables

**Table 1:** Summary of the Empirical Evidence by Model Segment.

Segment	Previous Evidence	Design and Sample	Limitation Addressed by This Study
AIL → RAI (H1)	Espinoza Vidaurre et al. (2025); Čolakovac et al. (2026); Park (2025)	SEM, *n* = 406, Peru; survey, *n* = 91; review of 51 studies	Measures attitudes or self-reported competence, not actual responsible behavior, with distinct dimensions
AIL → SRL (H2)	J. Wu et al. (2026); Acosta-Enriquez et al. (2025a); González-Prida et al. (2024)	Meta-analysis of 28 studies; PLS-SEM, *n* = 482, Peru; correlational, *n* = 84, Peru	Evaluates interventions or self-efficacy, not literacy as a multidimensional construct
SRL → RAI (H3)	Xu et al. (2025); Jiang and Xu (2026); Vereau Amaya et al. (2025)	Quasi-experiment, *n* = 68; experiment, *n* = 148; correlational, *n* = 200, Peru	Measures cognitive or affective outcomes, not responsible behavior
AIL → SRL → RAI (H4)	No prior comparison	Not applicable	Mediation has been identified (Vereau Amaya et al., 2025; García Castro et al., 2026) but not modeled
TSS × first stage (H5)	Marquez-Yauri et al. (2026); Yang and Tang (2026)	PLS-SEM, *n* = 340, Peru; PLS-SEM, *n* = 649, China	Analogous moderation not supported; interaction terms on observed scores
TSS × second stage (H6)	Cajas Bravo et al. (2025); Acosta-Enriquez et al. (2025b)	Multiple baseline design, *n* = 8, Peru; PLS-SEM, *n* = 876, Peru	Technostress is modeled as an antecedent or mediator, not as a condition of the behavior
TSS × direct path (H7)	Marquez-Yauri et al. (2026); Vargas-Bejarano et al. (2025)	PLS-SEM, *n* = 340, Peru; ordinal regression, *n* = 258, Peru	Resistance to use is examined, not normative quality of use
Moderated mediation (H8)	No prior comparison	Not applicable	No study estimates conditional indirect effects with latent variables

*Note.* AIL = artificial intelligence literacy; SRL = self-regulated learning; TSS = technostress; RAI = responsible use of generative artificial intelligence.

**Table 2:** Sample Composition (*N* = 820).

Characteristic	Category	*n*	%
Gender	Female	460	56.1
	Male	350	42.7
	Prefers not to say	10	1.2
Age	M = 22.72, SD = 2.92, range 18 to 34		
Academic cycle	I–II	128	15.6
	III–IV	188	22.9
	V–VI	197	24.0
	VII–VIII	179	21.8
	IX–X	128	15.6
Field of Study	Engineering	180	22.0
	Business	227	27.7
	Health Sciences	154	18.8
	Social Sciences	165	20.1
	Education	94	11.5
Type of university	Public	365	44.5
	Private	455	55.5
Region	Metropolitan Lima	482	58.8
	Other regions	338	41.2
Employment status	Full-time student	355	43.3
	Works part-time	301	36.7
	Works full-time	164	20.0
Frequency of generative AI use	No current academic use	81	9.9
	Rarely	194	23.7
	Occasionally or monthly	234	28.5
	Weekly	204	24.9
	Daily	107	13.0
Primary tool	ChatGPT	419	51.1
	Gemini	140	17.1
	Claude	90	11.0
	Copilot	76	9.3
	DeepSeek	53	6.5
	Other	42	5.1
Training received on AI	None	249	30.4
	Self-taught	260	31.7
	One-time workshop	231	28.2
	Institutional program	80	9.8
Institutional Policy on AI	None	306	37.3
	Not aware of it	374	45.6
	It does exist	140	17.1

*Note.* Percentages are calculated based on the total sample size and may not add up to 100 due to rounding. Frequency of generative AI use refers to use for academic work during the survey period and is distinct from the eligibility screening item, which asked about prior experience with at least one generative AI tool at any point (Section 2.2). The 81 cases in the “no current academic use” category therefore met the inclusion criterion; a sensitivity analysis excluding them is reported in Section 3.8.

**Table 3:** Operationalization of the Constructs.

Construct	Dimension	Code	k	Anchor Item (Adapted Example)	Source of Adaptation
AI Literacy (AIL, independent variable)	Awareness	AILCON	3	I identify the AI technology used by the apps and products I use.	B. Wang et al. (2023)
	Use	AILUSO	3	I use AI applications with ease to support my daily academic tasks.	B. Wang et al. (2023)
	Evaluation	AILEVA	3	I can assess the capabilities and limitations of an AI application after using it for a while.	B. Wang et al. (2023)
	Ethics	AILETI	3	I remain vigilant against the misuse of AI technology.	B. Wang et al. (2023)
Self-Regulated Learning (SRL, mediator)	Goal setting	SRLMET	3	I set short- and long-term goals for my courses.	Barnard et al. (2009); Pinto Santubera et al. (2020)
	Learning Strategies	SRLEST	3	I prepare my questions before consulting a reference source.	Barnard et al. (2009); Zimmerman (2002)
	Time Management	SRLTIE	3	I try to study at a set time and stick to it.	Barnard et al. (2009)
	Self-Assessment	SRLAUT	3	I summarize what I’ve learned to check how much I’ve understood.	Barnard et al. (2009)
Technostress (TSS, moderator)	Skill-demand mismatch	TSSHAB	4	I find it difficult to meet the technological demands of my courses with the skills I have.	X. Wang and Li (2019); Verde-Avalos et al. (2025)
	Needs–Resources Mismatch	TSSNEC	4	I am uncomfortable with the constant presence of technology in all aspects of my studies.	X. Wang et al. (2020); Verde-Avalos et al. (2025)
	Person-to-person mismatch	TSSPER	3	I don’t receive the support I need from my peers to use these technologies.	X. Wang and Li (2019); Verde-Avalos et al. (2025)
Responsible use of generative AI (RAI, dependent variable)	Normative integrity	RAIINT	4	I follow my university’s rules regarding the use of AI in graded assignments.	Original work based on Chan (2023) and UNESCO (2023)
	Content verification	RAIVER	4	I cross-check the information provided by an AI tool with other sources before using it.	Compiled by the author based on UNESCO (2023)
	Authorship and transparency	RAIAUT	4	I disclose which part of my work was supported by an AI tool.	Adapted from Chan (2023)

*Note.* k = number of items. All items were answered on a five-point Likert scale. The statements in the anchor column are representative examples; the complete instrument is provided in the Supplementary Material (Table S4).

**Table 4:** Correspondence between Hypotheses and Statistical Tests.

Hypothesis	Parameter	Test to Test It
H1	*c′*_1_	Direct structural coefficient in the mediation model using bootstrap and in the LMS model
H2	*a*_1_	Structural coefficient in the mediation model with bootstrap and in the LMS model
H3	*b*_1_	Structural coefficient in the mediation model with bootstrap and in the LMS model
H4	*a*_1_·*b*_1_	Indirect effect with bias-corrected bootstrap CI (10,000 resamples)
H5	*a*_3_	Latent interaction AIL × TSS on the mediator (XWITH)
H6	*b*_2_	Latent interaction SRL × TSS on the dependent variable (XWITH)
H7	*c′*_3_	Latent interaction between AIL and TSS on the dependent variable (XWITH)
H8	Δ ind	Difference between conditional indirect effects and the Johnson–Neyman region of significance

*Note.* AIL = artificial intelligence literacy; SRL = self-regulated learning; TSS = technostress; *c′* = direct effect; *a*·*b* = indirect effect; Δ ind = difference between conditional indirect effects; LMS = latent moderated structural equations; XWITH = Mplus operator for latent interaction terms; CI = confidence interval. Bootstrap confidence intervals are bias-corrected and based on 10,000 resamples; the conditional effects of the LMS model use delta-method intervals, as resampling is not compatible with numerical integration.

**Table 5:** Comparison of Measurement Models.

Model	par.	χ^2^	df	CFI	TLI	RMSEA	SRMR
Fourteen correlated dimensions	326	896.61	943	1.000	1.001	0.000	0.018
Second order (reference)	255	928.65	1014	1.000	1.002	0.000	0.020
Four factors at the item level	241	1395.11	1028	0.991	0.990	0.021	0.026
One-way	235	19,105.26	1034	0.549	0.529	0.146	0.151
Four constructs regarding dimensions	48	49.27	71	1.000	1.004	0.000	0.010

*Note: N* = 820. par. = number of free parameters. The first four models were estimated to use WLSMV on the 47 items treated as ordinal; the last model was estimated to use robust maximum likelihood on the 14 dimension scores. The χ^2^ statistics for WLSMV and MLR cannot be compared directly; therefore, the comparison is based on incremental and absolute indices. All *p*-values for the chi-square contrast are <0.001 except for the first two models and the last one, where they exceed 0.85.

**Table 6:** Reliability and Convergent Validity by Dimension and by Construct.

Scale	Dimension	k	α	CR	AVE	Range of λ	γ
AIL. AI Literacy							
AILCON	AI Awareness	3	0.820	0.854	0.661	0.777–0.858	0.956
AILUSO	Use of AI	3	0.846	0.877	0.704	0.819–0.857	0.944
AILEVA	AI Evaluation	3	0.830	0.861	0.674	0.770–0.858	0.925
AILETI	Ethics of AI	3	0.817	0.850	0.654	0.778–0.835	0.925
Total AIL	Second-order construct	12	0.937	0.946	0.592	√AVE = 0.769	-
SRL. Self-Regulated Learning							
SRLMET	Goal Setting	3	0.834	0.864	0.679	0.790–0.855	0.947
SRLEST	Learning Strategies	3	0.832	0.863	0.678	0.808–0.850	0.945
SRLTIE	Time Management	3	0.806	0.836	0.630	0.758–0.818	0.951
SRLAUT	Self-Assessment	3	0.836	0.866	0.682	0.790–0.855	0.912
Total SRL	Second-order construct	12	0.937	0.945	0.588	√AVE = 0.767	-
TSS. Technostress							
TSSHAB	Skill–Demand Mismatch	4	0.869	0.894	0.679	0.785–0.858	0.944
TSSNEC	Mismatch between needs and resources	4	0.858	0.884	0.655	0.780–0.860	0.916
TSSPER	Person-to-person mismatch	3	0.813	0.845	0.646	0.774–0.820	0.893
Total TSS	Second-order construct	11	0.927	0.933	0.561	√AVE = 0.749	-
RAI. Responsible Use of Generative AI							
RAIINT	Regulatory Integrity	4	0.857	0.885	0.658	0.788–0.827	0.959
RAIVER	Content Verification	4	0.860	0.886	0.661	0.770–0.846	0.929
RAIAUT	Authorship and Transparency	4	0.856	0.886	0.660	0.781–0.844	0.921
Total RAI	Second-order construct	12	0.935	0.943	0.579	√AVE = 0.761	-

*Note.* k = number of items; α = Cronbach’s alpha; CR = composite reliability; AVE = average variance extracted; λ = standardized factor loading of the item; γ = loading of the dimension on the second-order construct. CR and AVE were calculated based on the fully standardized loadings of the WLSMV model. Criteria: CR ≥ 0.70 and AVE ≥ 0.50.

**Table 7:** Discriminant validity: Fornell–Larcker criterion and heterotrait–monotrait ratio.

Construct	1. AIL	2. SRL	3. TSS	4. RAI
Latent correlations and AVE root on the diagonal				
AIL	0.769			
SRL	0.625	0.767		
TSS	−0.207	−0.099	0.749	
RAI	0.565	0.616	−0.296	0.761
Heterotrait–monotrait ratio				
AIL	-			
SRL	0.608	-		
TSS	0.201	0.096	-	
RAI	0.547	0.597	0.284	-

*Note. N* = 820. AIL = artificial intelligence literacy; SRL = self-regulated learning; TSS = technostress; RAI = responsible use of generative AI. The latent correlations are derived from the second-order model; the heterotrait–monotrait ratio was calculated based on the polychoric correlations of the 47 items. Criteria: The square root of the AVE must exceed any correlation of the construct with the others, and the heterotrait–monotrait ratio must be below 0.85. All correlations are significant, *p* < 0.001, except for SRL-TSS, *p* = 0.014.

**Table 8:** Test of Measurement Invariance by Gender and University Type.

Model	par.	χ^2^	df	CFI	RMSEA	SRMR	ΔCFI	Δχ^2^ (gl), *p*
Gender								
Configural	482	2433.06	2056	0.990	0.021	0.035	-	-
Metric	439	2467.85	2099	0.991	0.021	0.036	0.001	39.30 (43), 0.632
Scale	302	2584.20	2236	0.991	0.020	0.036	0.000	110.83 (137), 0.951
Type of university								
Configural	482	2414.24	2056	0.991	0.021	0.035	-	-
Metric	439	2458.02	2099	0.991	0.020	0.035	0.000	50.96 (43), 0.189
Scale	302	2610.23	2236	0.991	0.020	0.035	0.000	172.95 (137), 0.020

*Note.* Model estimated using the Mplus command *MODEL = CONFIGURAL METRIC SCALAR*, with the WLSMV estimator and theta parameterization. Δχ^2^ corresponds to the adjusted difference test compared to the immediately preceding model. Practical criterion: ΔCFI ≤ 0.010 and ΔRMSEA ≤ 0.015. Cases that did not report gender (*n* = 10) were excluded from the comparison by gender.

**Table 9:** Descriptive statistics and correlations among latent constructs.

Construct	M	SD	1	2	3	4
1. AI Literacy	3.46	0.82	-			
2. Self-Regulated Learning	3.26	0.84	0.625	-		
3. Technostress	2.77	0.83	−0.207	−0.099	-	
4. Responsible Use of Generative AI	3.45	0.80	0.565	0.616	−0.296	-

*Note. N* = 820. *M* and *SD* correspond to the observed composite scores on a scale of 1 to 5. The correlations are between latent constructs and are derived from the second-order model. All are significant, *p* < 0.001, except for the correlation between self-regulated learning and technostress, *p* = 0.014.

**Table 10:** Coefficients of the mediation model.

Path	b	SE	β	*p*	95% CI
SRL ← AIL	0.510	0.029	0.626	<0.001	[0.454, 0.567]
SRL ← TSS	0.025	0.028	0.031	0.370	[−0.029, 0.081]
RAI ← AIL	0.189	0.034	0.243	<0.001	[0.123, 0.258]
RAI ← SRL	0.425	0.042	0.445	<0.001	[0.346, 0.509]
RAI ← TSS	−0.152	0.026	−0.195	<0.001	[−0.203, −0.101]
Decomposition of the effect of AIL on RAI					
Total effect	0.406	0.028	0.522	<0.001	[0.351, 0.461]
Indirect effect via SRL	0.217	0.023	0.278	<0.001	[0.175, 0.265]
Direct effect	0.189	0.034	0.243	<0.001	[0.123, 0.258]

*Note. N* = 820. Maximum likelihood estimation. *b* = unstandardized coefficient; *SE* = standard error; β = fully standardized coefficient. Confidence intervals are bias-corrected bootstrap intervals based on 10,000 resamples and correspond in all cases to the unstandardized metric, i.e., they bound *b* and not β. AIL = artificial intelligence literacy; SRL = self-regulated learning; TSS = technostress; RAI = responsible use of generative AI. *R*^2^ = 0.385 for SRL and 0.466 for RAI.

**Table 11:** Structural coefficients and conditional effects of the moderated mediation model.

Parameter	Estimate	SE	z	*p*	95% CI
Structural coefficients					
*a*_1_ SRL ← AIL	0.513	0.028	18.12	<0.001	[0.458, 0.568]
*a*_2_ SRL ← TSS	0.025	0.028	0.89	0.373	[−0.030, 0.080]
*a*_3_ SRL ← AIL × TSS	−0.020	0.026	−0.77	0.440	[−0.071, 0.031]
*c′*_1_ RAI ← AIL	0.182	0.033	5.59	<0.001	[0.117, 0.247]
*b*_1_ RAI ← SRL	0.435	0.038	11.36	<0.001	[0.361, 0.509]
*c′*_2_ RAI ← TSS	−0.150	0.024	−6.18	<0.001	[−0.197, −0.103]
*c′*_3_ RAI ← AIL × TSS	0.046	0.034	1.34	0.182	[−0.021, 0.113]
*b*_2_ RAI ← SRL × TSS	−0.211	0.040	−5.24	<0.001	[−0.289, −0.133]
Conditional indirect effect of AIL on RAI through SRL					
Low technostress (−1 SD)	0.344	0.036	9.67	<0.001	[0.273, 0.415]
Mean technostress	0.223	0.022	10.23	<0.001	[0.180, 0.266]
High technostress (+1 SD)	0.111	0.030	3.73	<0.001	[0.052, 0.170]
Difference (high − low)	−0.233	0.048	−4.88	<0.001	[−0.327, −0.139]
Conditional direct effect of AIL on RAI					
Low technostress (−1 SD)	0.137	0.046	2.95	0.003	[0.047, 0.227]
Average technostress	0.182	0.033	5.59	<0.001	[0.117, 0.247]
High technostress (+1 SD)	0.228	0.048	4.72	<0.001	[0.134, 0.322]
Difference (high − low)	0.092	0.069	1.34	0.182	[−0.043, 0.227]

*Note. N* = 820. Robust maximum likelihood estimation with Monte Carlo integration (5000 points). AIL and TSS were scaled by fixing their latent variance at 1, so that moderator levels are expressed in standard deviations. The confidence intervals are symmetric and derived from the delta method, since Mplus does not support bootstrapping with numerical integration. *SD* = standard deviation.

**Table 12:** Results of the Hypothesis Testing.

Hypothesis	Statement	Result	Evidence
H1	AI literacy positively predicts the responsible use of generative AI	Supported	β = 0.243, *p* < 0.001
H2	AI literacy positively predicts self-regulated learning	Supported	β = 0.626, *p* < 0.001
H3	Self-regulated learning positively predicts responsible use	Supported	β = 0.445, *p* < 0.001
H4	Self-regulated learning mediates the relationship between AI literacy and responsible use	Supported	β = 0.278, 95% CI [0.226, 0.337]
H5	Technostress moderates the effect of literacy on self-regulated learning	Not supported; no significant moderation detected	a_3_ = −0.020, *p* = 0.440
H6	Technostress moderates the effect of self-regulated learning on responsible use	Supported	*b*_2_ = −0.211, *p* < 0.001
H7	Technostress moderates the direct effect of AI literacy on responsible use	Not supported; no significant moderation detected	c′_3_ = 0.046, *p* = 0.182
H8	The indirect effect is conditional on the level of technostress	Supported	Δ = −0.233, *p* < 0.001

*Note.* Hypotheses H1 through H4 were tested in the mediation model using bootstrap, and hypotheses H5 through H8 were tested in the moderated mediation model with latent interaction. Δ = difference between the indirect effect at high technostress and at low technostress.

**Table 13:** Comparison of the Findings with Previous Literature.

Hypothesis	Converges or Diverges with	Explanation Provided
H1	Converges with Espinoza Vidaurre et al. (2025)	The ethical dimension is part of the construct of literacy
H2	Consistent with J. Wu et al. (2026) and Acosta-Enriquez et al. (2025a)	Competence may support planning rather than replace it
H3	Consistent with Xu et al. (2025) and Jiang and Xu (2026)	Self-observation and judgment against standards are the operations required by responsible use
H4	Original contribution; consistent with Fan et al. (2025)	Dependent behavior combines low-cost acts and deliberate acts
H5	Consistent with Marquez-Yauri et al. (2026), H. Wu et al. (2026), and Di Stefano et al. (2025)	Possible cancellation of challenge and hindrance stressors under aggregation (candidate explanation, not tested here)
H6	Consistent with Kong et al. (2026) and Lallmahomed (2025)	Verification and declaration are costly actions that resource conservation predicts will be cut first
H7	Consistent with Marquez-Yauri et al. (2026); inconsistent with Türk et al. (2025) and Duong et al. (2025)	The direct path accounts for low-cost regulatory compliance; the divergences measured technological anxiety regarding acceptance and well-being outcomes, not regarding regulated behavior
H8	Original contribution; contrasts with Di Stefano et al. (2025)	Second-stage moderation extends to the indirect effect; the associated region-of-significance boundary is an estimate, not a threshold

*Note.* J-N = Johnson–Neyman. The decisions are based on the models reported in Section 3.

## Data Availability

The data presented in this study are available upon request from the corresponding author. The data are not publicly available because participants consented only to anonymous and aggregated analysis.

## Supplementary Materials

The following supporting information can be downloaded at: https://www.mdpi.com/article/10.3390/ejihpe16090143/s1, Table S1: Assessment criteria and decision thresholds; Table S2: Descriptive statistics and standardized factor loadings of the items; Table S3: Robustness of the moderated mediation model with respect to control covariates; Table S4: Full instrument: statements for the 47 items by dimension.
