# Supplementary material for: Artificial Intelligence Literacy and the Responsible Use of Generative AI Among University Students in Peru: The Mediating Role of Self-Regulated Learning and the Moderating Effect of Technostress
Source: Eur J Investig Health Psychol Educ. 2026 Sep 17;16(9):143. doi: 10.3390/ejihpe16090143 (PMC13605633; doi:10.3390/ejihpe16090143)
Supplement: Supplementary file 1 [file ejihpe-16-00143-s001.zip › ejihpe-4525417-supplementary.pdf]

Supplementary Material

The following tables are provided as online supplementary material and are cited in the text as Table S1, Table S2, Table S3, and Table S4.

Table S1

Assessment criteria and decision thresholds

Criterion	Threshold	Source
CFI, TLI	≥ .95 acceptable; ≥ .90 minimum	Hu and Bentler (1999); Marsh et al. (2004)
RMSEA	≤ .06 acceptable; ≤ .08 minimum	Hu and Bentler (1999)
SRMR	≤ .08	Hu and Bentler (1999)
Standardized factor loading	≥ .70 desirable; ≥ .50 minimum	Hair et al. (2019)
Reliability (α, ω, CR)	≥ .70	Cronbach (1951); McDonald (1999); Jöreskog (1971)
Extracted mean variance	≥ .50	Fornell and Larcker (1981)
Discriminant validity (Fornell-Larcker)	√AVE > correlation with any other construct	Fornell and Larcker (1981)
Discriminant validity (HTMT)	< .85	Henseler et al. (2015); Voorhees et al. (2016)
Measurement invariance	ΔCFI ≤ .010 and ΔRMSEA ≤ .015	Cheung and Rensvold (2002); Chen (2007)
Common-method bias	Method factor variance < 25%	Fuller et al. (2016); Liang et al. (2007)
Indirect effect	95% corrected bootstrap CI excluding zero	Preacher and Hayes (2008)
Latent interaction	Significant scaled log-likelihood difference, with lower AIC and BIC	Satorra and Bentler (2010); Klein and Moosbrugger (2000)
Conditional effect	95% CI using the delta method that excludes zero	Maslowsky et al. (2015)

Table S2

Descriptive statistics and standardized factor loadings of the items

Item	M	SD	Skewness	Kurtosis	λ
AIL. AI Awareness					
AIL1	3.65	1.04	−0.36	−0.56	.858
AIL2	3.57	1.04	−0.30	−0.60	.802
AIL3	3.51	1.04	−0.29	−0.57	.777
AIL. Use of AI					
AIL4	3.42	1.07	−0.24	−0.67	.857
AIL5	3.34	1.09	−0.14	−0.70	.841
AIL6	3.45	1.08	−0.28	−0.64	.819

Item	M	SD	Skewness	Kurtosis	λ
AIL. AI Assessment					
AIL7	3.34	1.09	−0.24	−0.59	.833
AIL8	3.27	1.08	−0.20	−0.61	.770
AIL9	3.44	1.04	−0.22	−0.59	.858
AIL. Ethics of AI					
AIL10	3.56	1.06	−0.34	−0.57	.813
AIL11	3.47	1.03	−0.25	−0.53	.835
AIL12	3.49	1.05	−0.20	−0.65	.778
SRL. Goal Setting					
SRL1	3.32	1.09	−0.16	−0.67	.826
SRL2	3.22	1.11	−0.12	−0.69	.790
SRL3	3.37	1.08	−0.20	−0.61	.855
SRL. Learning Strategies					
SRL4	3.27	1.04	−0.07	−0.58	.812
SRL5	3.32	1.08	−0.26	−0.49	.850
SRL6	3.17	1.07	−0.16	−0.60	.808
SRL. Time Management					
SRL7	3.15	1.12	−0.06	−0.65	.818
SRL8	3.09	1.10	−0.07	−0.73	.758
SRL9	3.28	1.09	−0.17	−0.64	.804
SRL. Self-Assessment					
SRL10	3.40	1.09	−0.25	−0.61	.855
SRL11	3.30	1.10	−0.21	−0.62	.832
SRL12	3.18	1.10	−0.14	−0.56	.790
TSS. Skills-Demands Mismatch					
TSS1	2.79	1.08	0.13	−0.60	.858
TSS2	2.73	1.10	0.14	−0.65	.785
TSS3	2.90	1.11	0.07	−0.62	.858
TSS4	2.78	1.09	0.09	−0.64	.792
TSS. Mismatch between needs and resources					
TSS5	2.96	1.10	0.00	−0.68	.804
TSS6	2.87	1.09	0.12	−0.64	.860
TSS7	2.91	1.09	0.01	−0.70	.780
TSS8	2.77	1.07	0.14	−0.58	.792
TSS. Person-to-person mismatch					
TSS9	2.56	1.09	0.32	−0.57	.820

Item	M	SD	Skewness	Kurtosis	λ
TSS10	2.52	1.08	0.25	−0.65	.816
TSS11	2.68	1.08	0.18	−0.67	.774
RAI. Regulatory Integrity					
RAI1	3.58	1.05	−0.41	−0.45	.827
RAI2	3.52	1.06	−0.32	−0.60	.788
RAI3	3.59	1.04	−0.31	−0.62	.819
RAI4	3:40	1.05	−0.23	−0.57	.811
RAI. Content Verification					
RAI5	3.37	1.09	−0.16	−0.69	.770
RAI6	3.23	1.09	−0.09	−0.72	.846
RAI7	3.33	1.05	−0.14	−0.61	.813
RAI8	3.45	1.06	−0.22	−0.70	.822
RAI. Authorship and Transparency					
RAI9	3.53	1.03	−0.23	−0.60	.844
RAI10	3.57	1.01	−0.19	−0.72	.789
RAI11	3.39	1.04	−0.14	−0.58	.833
RAI12	3.46	1.06	−0.25	−0.62	.781

Note. N = 820. Likert-type scale ranging from 1 (*strongly disagree*) to 5 (*strongly agree*). λ = fully standardized factor loading in the first-order model of 14 correlated dimensions, estimated using WLSMV on polychoric correlations. AIL = artificial intelligence literacy; SRL = self-regulated learning; TSS = technostress; RAI = responsible use of generative AI. All loadings are significant, $p < .001$.

Table S3

Robustness of the moderated mediation model with respect to control covariates

Parameter	Without controls	Controls on M and Y	Full controls
Structural coefficients			
a_1 SRL ← AIL	.513***	.497***	.427***
a_2 SRL ← TSS	.025	.026	.027
a_3 SRL ← AIL × TSS	−.020	−.017	−.013
c'_1 RAI ← AIL	.182***	.185***	.181***
b_1 RAI ← SRL	.435***	.441***	.355***
c'_2 RAI ← TSS	−.150***	−.146***	−.094**
c'_3 RAI ← AIL × TSS	.046	.044	.037
b_2 RAI ← SRL × TSS	−.211***	−.212***	−.205***
Conditional indirect effects			
Low technostress (−1 SD)	.344***	.336***	.247***

Parameter	Without controls	Controls on M and Y	Full controls
Moderate technostress	.223***	.219***	.151***
High technostress (+1 SD)	.111***	.110***	.062
Difference (high − low)	−.233***	−.226***	−.185***
Fit			
Unrestricted parameters	51	69	86
AIC	22,521.5	22,548.4	22,316.8
BIC	22,761.7	22,873.3	22,721.8

Note. *N* = 820. Covariates: gender, age, academic cycle, type of university, employment status, frequency of generative AI use, training received, and existence of institutional policy. In the second specification, the covariates predict the mediator and the outcome; in the third, they also predict the two exogenous variables, such that the latent interaction is defined over the portions of *X* and *W* that are independent of sociodemographic composition. *SD* = standard deviation. * p < .05. ** p < .01. *** p < .001.

Table S4

Full instrument: statements for the 47 items by dimension

Code	*Dimension*	*Item*
AIL1	*AI Awareness*	*I can identify when an application or platform I use in my studies is powered by artificial intelligence.*
AIL2	*AI Awareness*	*I understand, in broad terms, how a generative AI tool generates its responses.*
AIL3	*AI Awareness*	*I can distinguish between what generative AI does well and what it still does poorly.*
AIL4	*Using AI*	*I am proficient in using the generative AI tools I need for my academic tasks.*
AIL5	*Use of AI*	*I write prompts that get the AI to deliver what I'm looking for.*
AIL6	*Using AI*	*I learn how to use a new AI tool without anyone explaining it to me.*
AIL7	*Evaluating AI*	*I evaluate whether the AI's response is appropriate for the academic purpose at hand.*
AIL8	*AI Evaluation*	*I identify errors or fabrications in the content produced by the AI.*
AIL9	*AI Evaluation*	*I compare answers from different tools or sources before deciding which one to use.*

Code	*Dimension*	*Item*
AIL10	*AI Ethics*	*I am aware of the risks of providing personal or third-party data to an AI.*
AIL11	*AI Ethics*	*I recognize the ethical dilemmas that arise when using AI in academic work.*
AIL12	*AI Ethics*	*I consider the consequences that my use of AI may have on other people.*
SRL1	*Goal Setting*	*I set specific goals before I begin studying a topic.*
SRL2	*Goal Setting*	*I set high standards for the quality of my academic work.*
SRL3	*Goal Setting*	*In addition to my long-term goals, I set short-term, daily, or weekly goals.*
SRL4	*Learning Strategies*	*I prepare my own questions or outlines to check whether I've understood the material.*
SRL5	*Learning Strategies*	*I change my study strategy when the one I'm using isn't working for me.*
SRL6	*Learning Strategies*	*I connect what I'm learning now with what I already know from other courses.*
SRL7	*Time Management*	*I spread out my study time throughout the week instead of leaving everything for the last minute.*
SRL8	*Time Management*	*I set aside fixed blocks of time in my schedule for studying, even though no one requires me to.*
SRL9	*Time Management*	*I meet the deadlines I set for myself to make progress on my work.*
SRL10	*Self-Assessment*	*I review the quality of my work before turning it in.*
SRL11	*Self-Assessment*	*After each assessment, I reflect on what worked for me and what didn't.*
SRL12	*Self-Assessment*	*I compare my actual performance with the goals I had set for myself.*
TSS1	*Skills—~~-~~Demand Mismatch*	*The digital tools required for my courses demand more skills than I have.*
TSS2	*Skills—~~-~~demands mismatch*	*I have a hard time keeping up with the constant changes in the platforms and apps we use.*

Code	*Dimension*	*Item*
TSS3	*Skills-demands mismatch*	*I feel that my technological knowledge falls short of what the courses require of me.*
TSS4	*Skills-demands mismatch*	*It takes me longer than my classmates to learn how to use new technology.*
TSS5	*Mismatch between needs and resources*	*The technology available at my university does not meet my needs for effective learning.*
TSS6	*Mismatch between needs and resources*	*The platforms I'm required to use make studying more difficult rather than easier.*
TSS7	*Mismatch between needs and resources*	*When something goes wrong, the technical support I receive is insufficient.*
TSS8	*Mismatch between needs and resources*	*The digital tools in my courses don't fit my learning style.*
TSS9	*Person-to-person mismatch*	*I feel like my classmates are better at using the course technologies than I am.*
TSS10	*Person-to-people mismatch*	*It makes me uncomfortable when others notice my difficulties with technology.*
TSS11	*Person-to-people mismatch*	*Compared to people in my academic circle, I'm falling behind in using new tools.*
RAI1	*Regulatory Integrity*	*I use generative AI only for assignments where the instructor or course policy allows it.*
RAI2	*Regulatory Integrity*	*I avoid presenting work produced primarily by AI as my own.*
RAI3	*Regulatory Integrity*	*Before using AI in a graded assignment, I familiarize myself with my university's rules on the matter.*
RAI4	*Regulatory Integrity*	*When I'm unsure whether a particular use of AI is permitted, I seek guidance before proceeding.*
RAI5	*Content Verification*	*I cross-check the data that the AI includes in its responses against reliable sources.*
RAI6	*Content Verification*	*I verify that the bibliographic references suggested by the AI actually exist.*
RAI7	*Content Verification*	*I correct any errors I find in the AI-generated material before incorporating it into my work.*

Code	*Dimension*	*Item*
RAI8	*Content verification*	*I take responsibility for the accuracy of what I submit, even if I created it with the help of AI.*
RAI9	*Authorship and Transparency*	*I disclose or cite the use of generative AI when it contributes to a work I submit.*
RAI10	*Authorship and Transparency*	*I rephrase the material I obtain from AI using my own words and ideas.*
RAI11	*Authorship and Transparency*	*I make the important decisions regarding the work myself, without delegating them to the AI.*
RAI12	*Authorship and Transparency*	*In my work, I clearly distinguish between my own contributions and those made by the AI.*

Note: AIL = artificial intelligence literacy; SRL = self-regulated learning; TSS = technostress; RAI = responsible use of generative artificial intelligence. All items were rated on a five-point Likert scale. The AIL items were adapted from B. Wang et al. (2023); the SRL items, from Barnard et al. (2009) and Zimmerman (2002); and the TSS items, from X. Wang and Li (2019) and X. Wang et al. (2020). The RAI items were developed in-house based on Chan (2023) and UNESCO (2023).
